# Genetics‐Based Targeting Strategies for Precise Neuromodulation

**DOI:** 10.1002/advs.202413817

**Published:** 2025-05-19

**Authors:** Yuyuan He, Zhidong Wei, Jianda Xu, Fei Jin, Tong Li, Lili Qian, Juan Ma, Weiying Zheng, Negar Javanmardi, Ting Wang, Kangjian Sun, Zhang‐Qi Feng

**Affiliations:** ^1^ School of Chemistry and Chemical Engineering Nanjing University of Science and Technology Nanjing 210094 P.R. China; ^2^ Department of Orthopedics Changzhou Hospital of Traditional Chinese Medicine Changzhou Hospital Affiliated to Nanjing University of Chinese Medicine Changzhou 213003 P. R. China; ^3^ State Key Laboratory of Digital Medical Engineering Southeast University Nanjing 210096 P.R. China; ^4^ The Fourth Affiliated Hospital of Nanjing Medical University Nanjing 210031 P. R. China

**Keywords:** chemogenetic, electrogenetic, magnetogenetic, mechanogenetic, optogenetics, precise neuromodulation, thermogenetic

## Abstract

Genetics‐based neuromodulation schemes are capable of selectively manipulating the activity of defined cell populations with high temporal–spatial resolution, providing unprecedented opportunities for probing cellular biological mechanisms, resolving neuronal projection pathways, mapping neural profiles, and precisely treating neurological and psychiatric disorders. Multimodal implementation schemes, which involve the use of exogenous stimuli such as light, heat, mechanical force, chemicals, electricity, and magnetic stimulation in combination with specific genetically engineered effectors, greatly expand their application space and scenarios. In particular, advanced wireless stimulation schemes have enabled low‐invasive targeted neuromodulation through local delivery of navigable micro‐ and nanosized stimulators. In this review, the fundamental principles and implementation protocols of genetics‐based precision neuromodulation are first introduced.The implementation schemes are systematically summarized, including optical, thermal, force, chemical, electrical, and magnetic stimulation, with an emphasis on those wireless and low‐invasive strategies. Representative studies are dissected and analyzed for their advantages and disadvantages. Finally, the significance of genetics‐based precision neuromodulation is emphasized and the open challenges and future perspectives are concluded.

## Graphical abstract

In this Review, the fundamental principles and implementation protocols of genetics‐based precision neuromodulation are first introduced. Then, wireless and low‐invasive strategies based on nano‐transducing materials are highlighted, along with a dissection and analysis of the strengths and weaknesses of representative studies. Finally, the significance of genetics‐based precision neuromodulation is emphasized, and the open challenges and future perspectives are concluded.

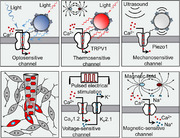

## 1 Introduction

The nervous system controls physiological processes through a sophisticated pattern of action potentials in neural networks that innervate all parts of the anatomy.^[^ ^1^, ^2^ ^]^ Complex mechanisms for the transmission and propagation of chemical and electrical signals between interconnected cell bodies, axons, dendrites, and synapses work together to process a wide variety of stimuli,^[^ ^3^, ^4^, ^5^ ^]^ including pain, pleasure, vision, somatic sensations, etc., as the basis for the regulation of organ function by the peripheral and central nervous systems.^[^ ^6^, ^7^ ^]^ Capabilities for modulating such neurological functions facilitate to establish an understanding of the principles underlying complex behaviors in animal species, and may address health conditions of significant societal burden, including neurological, neuropsychiatric, neuromuscular, and sensory disorders. Neuromodulation emerges as a rapidly advancing domain within the medical field,^[^ ^8^ ^]^ benefiting hundreds of thousands of patients afflicted with an array of neurological disorders. The market for neuromodulation is anticipated to ≈$7.8 billion by 2025.^[^ ^9^ ^]^ To date, the most widely adopted implementation utilizes electrical stimulators adapted from the architecture of cardiac pacemakers developed in the 1950s,^[^ ^10^ ^]^ in which electrical impulses are delivered to all cells within a millimeter scale through stimulating electrodes.^[^ ^11^ ^]^ However, to understand complex neural circuits and their relationship with specific behaviors and to precisely regulate neural circuits in underlying diseases to reduce post‐treatment side effects,^[^ ^12^, ^13^, ^14^ ^]^ requires spatiotemporally precise modulation of neuronal subtype activity at specific neural targets.^[^ ^15^, ^16^ ^]^ Genetics‐based neuromodulation technologies that allow highly precise manipulation of cellular physiology through the expression of specific response proteins or receptor proteins in defined cell populations, combined with physical field stimulation or subcellular targeting of micro‐ and nanoscale stimulators.^[^ ^17^, ^18^, ^19^ ^]^ Traditional neural stimulation techniques, such as deep brain stimulation (DBS),^[^ ^20^ ^]^ transcranial magnetic stimulation (TMS),^[^ ^21^ ^]^ and transcranial current stimulation (tCS), regulate brain activity through the targeted application of electrical currents or magnetic fields. DBS has been clinically proven in randomized trials to significantly improve symptoms in Parkinson's disease patients, including tremors, bradykinesia, rigidity, and axial symptoms.^[^ ^22^, ^23^ ^]^ TMS has been shown to be an effective and safe intervention for treatment‐resistant depression and other conditions,^[^ ^24^ ^]^ and when combined with brain mapping technologies, it aids in better understanding the interactions between different brain regions. Complementing TMS, tCS uses direct current to modulate brain activity by placing at least two surface electrodes on the scalp (one anode and one cathode) in its simplest configuration.^[^ ^25^ ^]^ Despite the effectiveness of these technologies in modulating neural activity, they also face several challenges. The primary issues include limited ability to selectively target specific neural circuits or cell types and difficulties achieving precise spatial resolution in certain brain regions. These problems mainly stem from the invasive implantation surgery required for DBS and the large‐sized stimulators that TMS and tCS need to make direct contact with the scalp.^[^ ^26^ ^]^ To overcome these limitations, researchers are exploring new methods and technologies to enhance the selectivity and precision of these neural stimulation techniques, thereby further optimizing their clinical application outcomes. Such methodologies offer three distinct advantages over conventional neuromodulation techniques. First, tailor‐made genetic tools allow precise control of specialized synaptic mechanisms, ensuring the fidelity of neuromodulation and facilitating multimodal neuromodulation. Second, the complex wiring of the nervous system is best understood by precisely modulating the functional connectivity of subpopulations of neurons; precise neuronal activation can reveal synaptic connections between two neuronal populations, while neuronal inhibition allows us to decipher the role of synapses in signal propagation, network oscillations, computation, and behavior. Third, unique manipulation schemes enable minimally invasive or even non‐invasive neuromodulation; wireless delivery of physical fields that can penetrate tissue non‐destructively facilitates safe neuromodulation.

**Figure 1:**
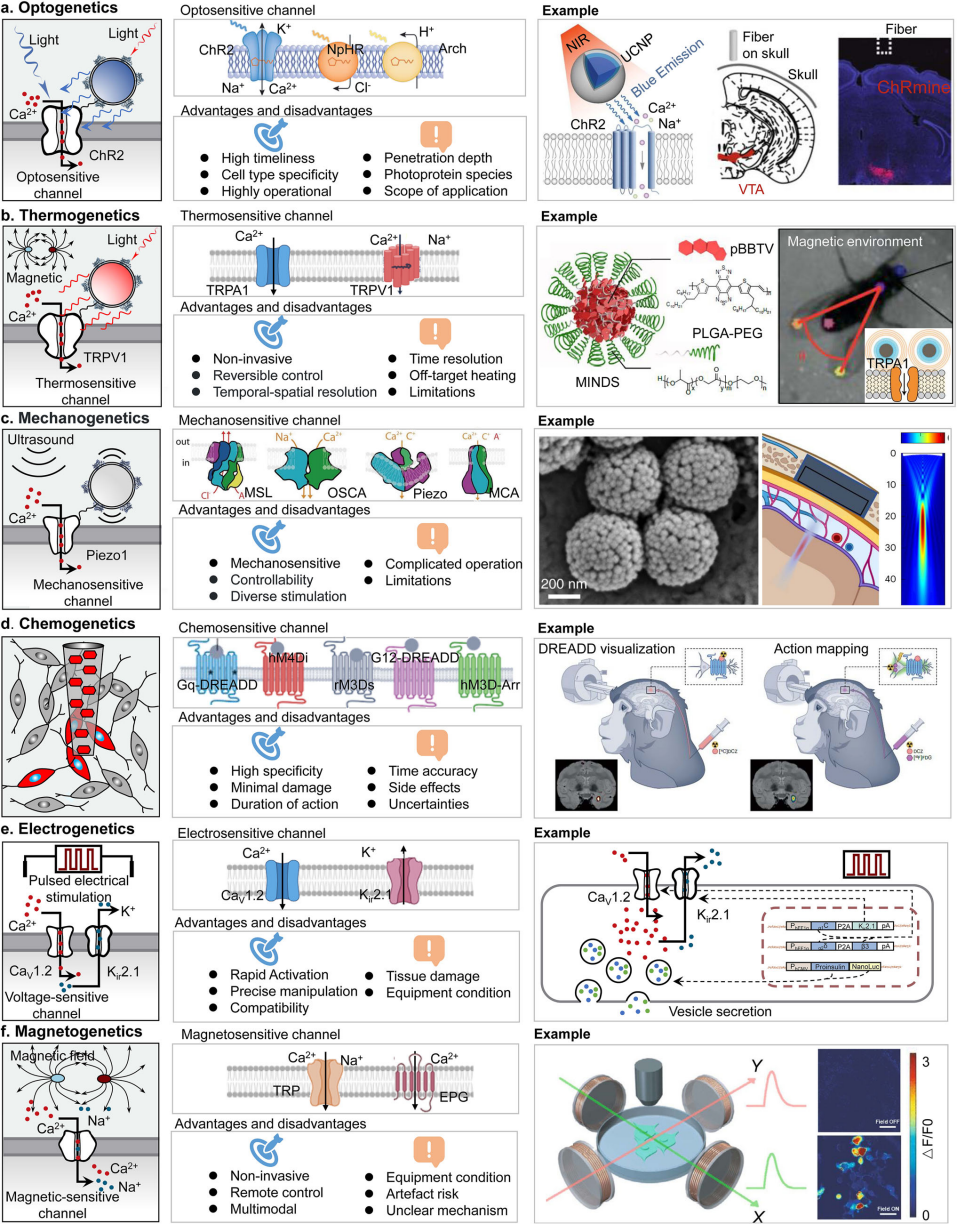
Summarized genetics‐based targeting strategies for precise neuromodulation. a) Optosensitive channel. Reproduced with permission.^[^ ^50^ ^]^ Copyright 2022, Spring Nature. Examples: Left, schematic principle of UCNPs (upconversion nanoparticles)‐mediated NIR (near‐infrared) upconversion optogenetics. Reproduced with permission.^[^ ^76^ ^]^ Copyright 2018, American Association for the Advancement of Science. Right, deep transcranial photoactivation. Reproduced with permission.^[^ ^241^ ^]^ Copyright 2021, Spring Nature. b) The part of thermogenetics Examples: Left, schematic showing the composition of MINDS (macromolecular infrared nanotransducers for deep‐brain stimulation). Reproduced with permission.^[^ ^242^ ^]^ Copyright 2022, Spring Nature. Right, activation of the alternating magnetic field (AMF) coil resulted in wing‐opening response due to TRPA1‐A channel activation by hysteretic heating of nearby nanoparticles. Reproduced with permission.^[^ ^243^ ^]^ Copyright 2022, Spring Nature. c) Mechanosensitive channel. Reproduced with permission.^[^ ^118^ ^]^ Copyright 2022, Elsevier. Examples: Left, the m‐Torquer (a magnetic torquer) is composed of assembled octahedral magnetic nanoparticles. Reproduced with permission.^[^ ^164^ ^]^ Copyright 2021, Spring Nature. Right, Sonogenetics using focused US beams for visual restoration through the intact dura mater. Reproduced with permission.^[^ ^244^ ^]^ Copyright 2023, Spring Nature. d) Chemosensitive channel. Examples: chemogenetics for cell‐type‐specific modulation of signalling and neuronal activity. Reproduced with permission.^[^ ^245^ ^]^ Copyright 2023, Spring Nature. e) Electrogenetics examples: schematic representation of the electrically inducible insulin secretion pathway.^44^. f) Magnetogenetics examples: Magnetogenetic activation of HEK‐293 cells by remote magnetic stimulation. Reproduced with permission.^[^ ^246^ ^]^ Copyright 2015, Elsevier.

In this review, we summarized the recent advances in genetics‐based targeting neuromodulation. First, we introduce the fundamental principles and implementation protocols of genetics‐based neuromodulation. Then, we systematically overviewed its implementation schemes based on physical field stimulation in the form of light, heat, mechanical force, chemicals, electricity, and magnetic stimulation focusing on those wireless and low‐invasive strategies, dissected and analyzed their advantages and disadvantages. Based on six emerging genetics‐based technologies, we have further summarized their respective advantages, disadvantages, and the involved protein channels, highlighting advanced breakthrough examples (**Figure** **1**). The performance of these in terms of spatial resolution, tissue penetration depth, target specificity, and sensitivity were also compared (**Table** **1**). Finally, we highlight the significance of genetics‐based precision neuromodulation, discuss their potential shortcomings and future prospects from the perspective of translational applications.

**Table 1:** Comparison of performance metrics of exogenous stimuli.

Cue	Penetration depth	Temporal resolution	Sensitivity	Target specificity	Refs.
Light	< 1 mm and at spatial resolution of <100 µm	< 1 ms	4–1000‐fold	High	[247, 248, 249]
Temperature	> 1 cm and at spatial resolution of ≈100 µm	In range of milliseconds to seconds	5–1800‐fold	Moderate	[250, 251]
Mechanical force	5mm	3.2 MHz	–	Low to moderate	[118, 160]
Chemical signal	–	<1 ms to mins	100–1000	Moderate	[252]
Voltage	–	–	–	–	
Magnetic	Unlimited by tissue (up to several cm)	10 ms to min	–	Moderate to High (with magnetic nanoparticles and engineered receptors)	[253]

## 2 Principles and Protocols of Genetics‐Based Targeting Neuromodulation Strategy

**Figure 2:**
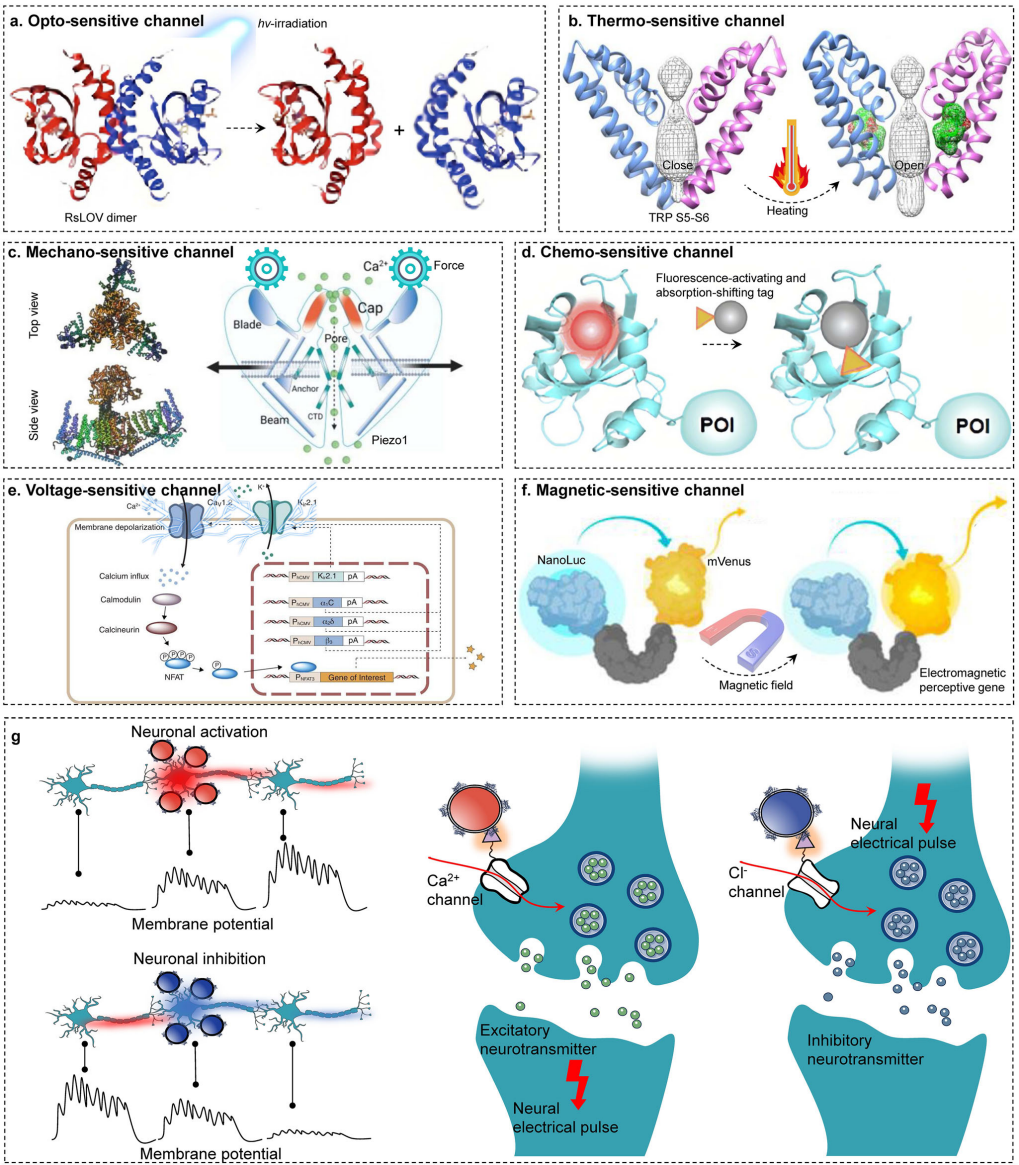
The foundational principles underlying genetics‐based targeted neuromodulation strategies. a) The blue‐light‐controlled light–oxygen–voltage (LOV) family of protein domains is widely used as PSMs (photosensory modules). Reproduced with permission.^[^ ^254^ ^]^ Copyright 2021, Spring Nature. b) Opening of the S6 gate in the S5–S6 structural domain after heating. Reproduced with permission.^[^ ^255^ ^]^ Copyright 2018, Spring Nature. c) Schematic diagram of the mechanotransduction and pore modules of the Piezol channel. Reproduced with permission.^[^ ^256^ ^]^ Copyright 2024, Elsevier. d) The stimuli‐responsive FAST system (srFAST) and the structural change of their ligand. Reproduced with permission.^[^ ^42^ ^]^ Copyright 2022, Royal Society of Chemistry. e) Calcium influx activates the calmodulin/calcineurin pathway, which leads to dephosphorylation of NFAT and its translocation to the nucleus, where it activates the NFAT‐sensitive promoter and triggers transgene expression. f) magnetic stimulation led to a conformational change in the EPG protein. Reproduced with permission.^[^ ^43^ ^]^ Copyright 2024, Frontiers. g) Cellular excitation and inhibition.

Throughout the natural world, spanning from plants and algae to bacteria, fungi, and advanced eukaryotic organisms,^[^ ^27^ ^]^ a plethora of specialized receptors have been developed. These receptors possess the unique ability to discern and react to external physical and chemical stimuli, thereby facilitating targeted electrophysiological adjustments in response to such cues.^[^ ^28^ ^]^ A notable illustration of this phenomenon is observed in the human retina, where photoreceptors are capable of detecting light and translating it into electrical signals conducive to vision. Similarly, cutaneous temperature receptors are adept at sensing variations in environmental temperatures, converting these perceptions into neural signals.^[^ ^29^, ^30^ ^]^ These naturally occurring sensory mechanisms present an invaluable resource for neuroscientists, offering the means for precise neuromodulation and significantly propelling advancements within the neuroscience domain. This section aims to elucidate the operational principles of these sensory receptors and explore their application in precise neurological interventions, as demonstrated in **Figure** **2**.

### 2.1 Artificially Manipulated Receptors

Receptors are fundamentally composed of a sensory module and an effector domain.^[^ ^28^ ^]^ Upon stimulation by specific cues, the receptors within the sensing module respond to the stimulus and undergo chemical transformations or conformational changes. As a result, the effector initiates a cascade of biological responses, encompassing enzymatic processes or protein–protein interactions.^[^ ^31^ ^]^ Different types of receptors exhibit distinct mechanisms in signal transduction based on the characteristics of their sensing modules. In the case of photoreceptors, many photoreceptors are bound to chromophores that exhibit off‐domain electrons distributed in conjugated p‐electron systems that absorb light in the 300 to 800 nm range. The absorption of photons instigates primary photochemical reactions, leading to alterations in protein structure that extend to the relevant effector domains (Figure 2a).^[^ ^32^ ^]^ Through rational and random mutagenesis, domain swapping, or modular combination with other proteins, photoreceptors can be engineered to respond to different wavelengths of light and selectively allow cations or anions (such as Cl^−^, Na^⁺^, H^⁺^, K^⁺^) to pass.^[^ ^33^, ^34^ ^]^ This alters the membrane potential across the cell membrane, achieving selective excitation or inhibition of cells. Heat receptors are typically ion channels sensitive to temperature changes, such as TRPV1, TRPM8, and TRPA1. They utilize temperature‐induced conformational changes or alterations in physical properties to regulate cellular processes. For instance, proteins or RNA thermometers (RNATs) with temperature‐sensitive folding structures can trigger conformational changes at specific temperatures, thereby activating or inhibiting downstream biological responses, achieving precise control over cellular activity (Figure 2g).^[^ ^35^ ^]^ The TRP selectivity filter is formed by a pore loop between S5 and S6 (Figure 2b).^[^ ^36^, ^37^, ^38^ ^]^ The Piezo1 channel features an extended‐arm trimeric bowl‐like structure.^[^ ^39^ ^]^ When force is applied, Piezo1 flattens further and transitions to an open state. This mechanical deformation leads to the opening of the channel, allowing ions to flow through and initiate downstream signaling events (Figure 2c).^[^ ^39^, ^40^, ^41^ ^]^ Chemogenetics, on the other hand, can induce structural changes in ligands through corresponding stimuli. Figure 2d illustrates how FAST achieves this by genetically fusing with targetable peptides or proteins, rationally designing “caged” ligands derived from HBR (Figure 2d).^[^ ^42^ ^]^ Similar to these are electrical stimulation and magnetic stimulation (Figure 2e,f),^[^ ^43^, ^44^ ^]^ both of which achieve cellular activity modulation by artificially synthesizing receptors with varying cue responsiveness.

### 2.2 Execution of Artificial Receptor‐Mediated Precision Neuromodulation

**Figure 3:**
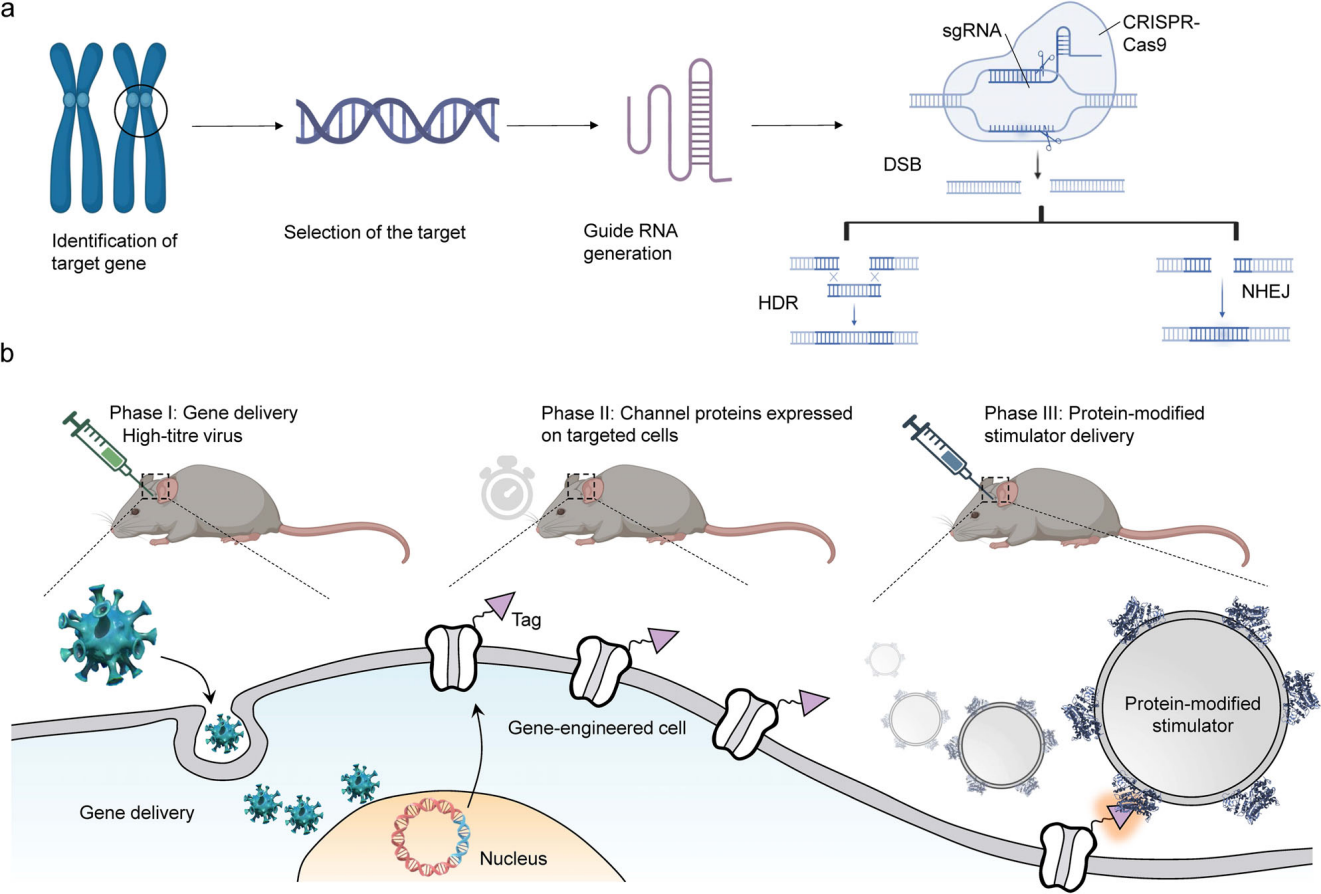
Execution of artificial receptor‐mediated precision neuromodulation. a) Designing suitable artificial receptors with gene editing. b) Delivery of engineered gene editing or regulatory tools to target neurons via specific vectors and expression in cells.

The development of artificial receptor‐mediated precision neuromodulation involves the integration of synthetic receptors with neuromodulatory techniques to facilitate targeted interventions within the nervous system. Initially, the process necessitates the identification of distinctive markers or genetic identifiers of the neuron or neural circuitry under consideration. This identification is achieved through the analysis of anatomical, functional, and transcriptomic datasets pertaining to the nervous system, enabling the selection of relevant markers or genes for targeting purposes. Following marker identification, the subsequent phase involves the design of gene editing or regulatory instruments tailored to the identified targets. Prominent gene editing methodologies,^[^ ^45^ ^]^ such as CRISPR–Cas9,^[^ ^46^, ^47^, ^48^ ^]^ are employed for the precise modification of gene sequences (**Figure** **3**a). Additionally, regulatory mechanisms, including enhancers and suppressors, are utilized to adjust gene expression levels to desired standards. The introduction of these genetic tools into target neurons is facilitated by gene delivery methods utilizing specific vectors. Vectors such as viral vectors, plasmids, and nanoparticles, with adeno‐associated virus (Adeno‐associated virus, AAV^[^ ^49^ ^]^) being a commonly preferred choice, are instrumental in this process. These vectors enable the incorporation and subsequent expression of gene editing or regulatory mechanisms within target cells (Figure 3b). The efficacy and impact of the introduced genetic modifications on target neurons are assessed through various molecular biology techniques, including immunohistochemistry and protein assays. These evaluations ascertain the successful expression and functionality of the gene editing or regulatory tools. Upon confirmation of effective gene tool expression, external stimuli can be applied to modulate neuronal activity. Simultaneously, it is necessary to employ appropriate genetic tools based on specific application requirements and objectives for neural modulation using light, heat, mechanical force, chemical signals, electrical stimuli, or magnetic fields. Once the editing or modulation tools are successfully expressed and functional, the activity of the targeted neuronal populations can be regulated using the corresponding stimuli (such as light, magnetic fields, electrical pulses, or chemical signals). Throughout this process, real‐time monitoring of neural activity and feedback information must be utilized to make adjustments and optimizations, ensuring optimal therapeutic outcomes.

## 3 Optogenetics

Optogenetic technologies control the activity of selected cells in highly heterogeneous tissues with temporal and spatial precision by utilizing a combination of natural and genetically encoded photoreceptors and light.^[^ ^50^, ^51^, ^52^ ^]^ The incorporation of regulatory elements such as promoters, enhancers, and specific targeting sequences into the DNA encoding for photoreceptors ensures the selective expression and subcellular localization within the host organism.^[^ ^50^ ^]^ This sophisticated approach permits the precise characterization and control of cellular functions, further propelling the field of precision neuromodulation forward.^[^ ^50^ ^]^ In this chapter, an examination of recent advancements in optogenetic actuators is provided, along with a comprehensive review of strategies for targeted light delivery and their respective scientific utilities. Additionally, the discourse extends to the exploration of the possibilities and constraints associated with the employment of optogenetic strategies for targeted neuromodulation, contemplating their prospective integration into clinical practice.

### 3.1 Genetically Engineered Light‐Controlled Switches for Cellular Activity

Photoreceptor proteins^[^ ^53^ ^]^ represent a pivotal class engaged in photoreception, facilitating the transduction of natural light signals into electrophysiological responses within cellular environments.^[^ ^54^ ^]^ Among the most notable are the retinal proteins of Drosophila and the channelrhodopsin (ChR) variants observed in Chlamydomonas reinhardtii.^[^ ^55^ ^]^ ≈900 ChR sequences have been cataloged, and the known family of ChRs includes cation‐conducting ChRs (CCRs) from the green alga Chlamydomonas reinhardtii and Cryptomeria japonica, and anion‐conducting ChRs (ACRs) from Cryptomeria japonica, Cryptomeria japonica, and Staphylosporidium japonica.^[^ ^56^ ^]^ CCRs predominantly facilitate the formation of cation channels, exhibiting a marked preference for protons, whereas their conductance of sodium ions is notably variable and divalent cations are largely ineffectual under typical physiological conditions.^[^ ^57^ ^]^ Recent macrogenomic screens have also identified a new class of potassium ion‐selective channels (KCRs).^[^ ^58^ ^]^ ACRs demonstrate selectivity towards a spectrum of anions, paralleling the functionality of human anion channels.^[^ ^59^, ^60^ ^]^ Recent macrogenomics has identified a new class of anion‐conducting and highly desensitized ChRs (MerMAID).^[^ ^56^ ^]^ Within host organisms, CCRs predominantly function as depolarizing agents via the photodriven influx of sodium (Na^+^) and calcium (Ca^2+^) ions, conversely, ACRs precipitate the influx of chloride ions, culminating in the reduction of cell membrane potential, and potentially leading to depolarization.^[^ ^61^, ^62^ ^]^ The recent identification of KCRs and MerMAID ChRs bearing cation and anion conduction capabilities, respectively, harbors significant potential for application. The existing spectrum of ChRs exhibits light sensitivities ranging from 445 to 610 nm, enabling the strategic combination of ChRs to simultaneously activate and inhibit cellular functions within the same experimental framework. However, when employing multiple ChRs in tandem, their proclivity for blue or UVA light absorption necessitates careful consideration to mitigate inadvertent activation of other ChRs.^[^ ^50^ ^]^

The employment of optogenetic instruments in gene targeting embodies a pivotal advancement towards achieving precise neuromodulation. Leveraging viral vector methodologies enables the efficient integration of photoreceptors into designated neuronal populations. One strategy involves transgenic expression, necessitating the maintenance of a singular animal lineage that exhibits optogenetic protein expression or the hybridization of two distinct animal lineages, with their first‐generation offspring manifesting the optogenetic protein gene across all cells expressing kinesin.^[^ ^63^ ^]^ Alternatively, the utilization of lentiviral or adeno‐associated virus (AAV) vectors, crafted to harbor optogenetic effectors, facilitates the expression of these effectors in specific cell types through the modification of promoter elements within the AAV construct. Notwithstanding the numerous benefits attributed to AAV vectors, they are not devoid of challenges, including constraints related to payload capacity and the selection of appropriate promoter elements.

### 3.2 Optogenetic‐Mediated Precision Neuromodulation

**Figure 4:**
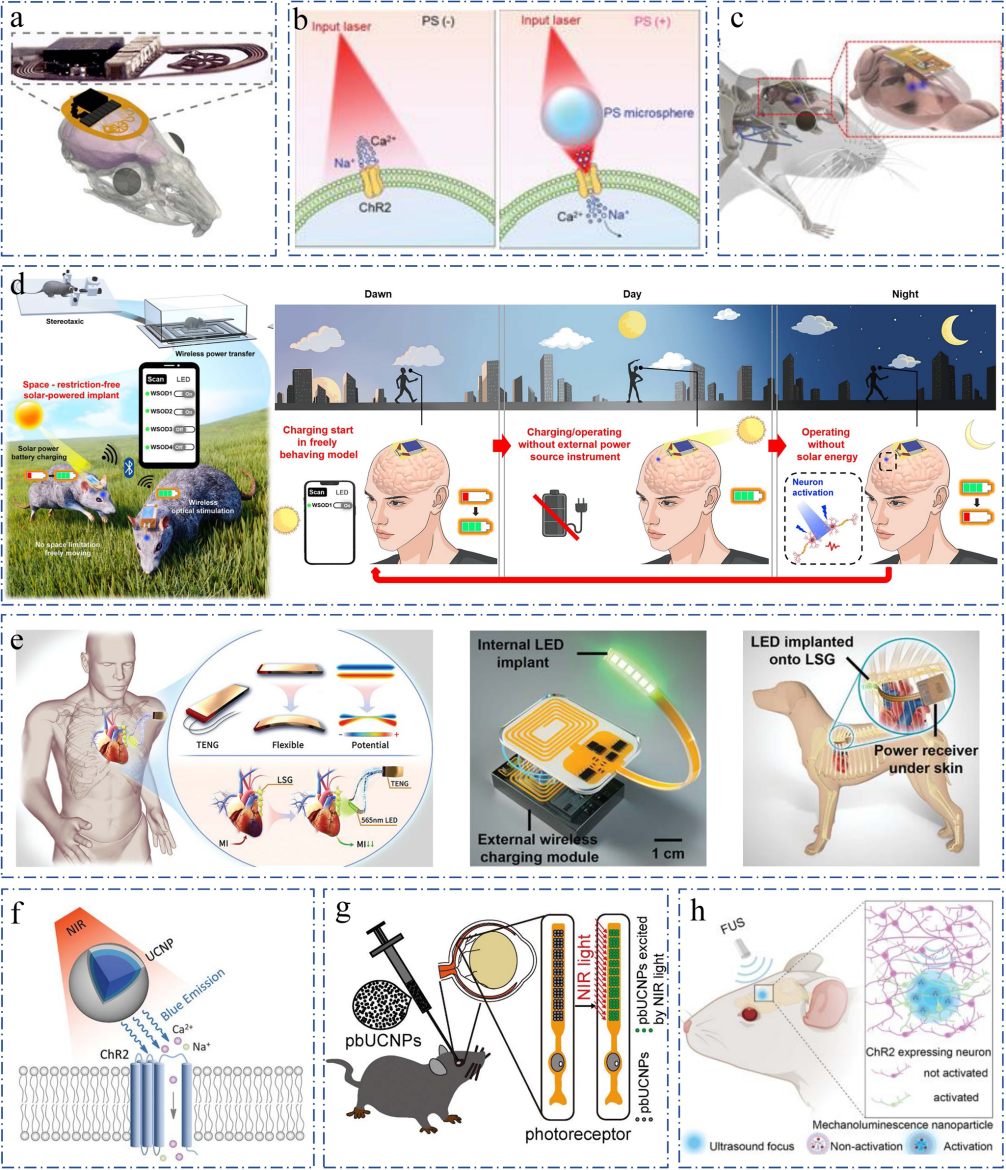
a) Wireless subcutaneous implantable device with capacitive energy storage for transcranial and remote optogenetics in freely moving animals. Reproduced with permission.^[^ ^68^ ^]^ Copyright 2021, National Academy of Sciences. b) Microsphere‐based optogenetics can successfully open ChR2 channels. Reproduced with permission.^[^ ^69^ ^]^ Copyright 2022, Wiley. c) Conceptual illustration of a wireless optoelectronic probe system subcutaneously implanted in a rodent head for control of neural circuits deep in the brain. The inset highlights conformal integration of the device with a rat brain. Reproduced with permission.^[^ ^70^ ^]^ Copyright 2021, Springer Nature. d) Left, conceptual illustration of wireless solar‐powered optogenetic device (WSOD) for a completely freely moving experiment. Right, conceptual illustration showing the cycles of wireless recharging and operating during outdoor activity for human brain application. After sunset charging starts, the device is fully charged without an external power source and spatial constraints. Energy storage enables humans to operate WSOD even at night. Reproduced with permission.^[^ ^71^ ^]^ Copyright 2023, AMER ASSOC ADVANCEMENT SCIENCE. e) Left, the optogenetic neuromodulation for cardioprotection by the self‐powered optical system is presented. Middle, schematic diagram and optical image of the implantable, battery‐free wireless optogenetic system. Right, diagram of the wireless LED device implantation in canines. Reproduced with permission.^[^ ^72^ ^]^ Copyright 2023, Wiley. f) Schematic principle of UCNPs (upconversion nanoparticles)‐mediated NIR (near‐infrared) upconversion optogenetics. Reproduced with permission.^[^ ^76^ ^]^ Copyright 2018, American Association for the Advancement of Science. g) Illustration of photoreceptor‐binding upconversion nanoparticles and generation of green light upon near‐infrared (NIR) light illumination. Reproduced with permission.^[^ ^77^ ^]^ Copyright 2019, Elsevier. h). FUS‐activated nanotransducers act as a wireless light source for spatiotemporal neuromodulation. Reproduced with permission.^[^ ^82^ ^]^ Copyright 2023, American Chemical Society.

Optogenetics is broadly applicable to preparations that are accessible to light such as cultured cells, tissue slices, and transparent organisms like zebrafish larvae, as well as the cortical surface of the mammalian brain, allowing for a wide range of flexibility in light delivery.^[^ ^64^, ^65^, ^66^ ^]^ For comprehensive neural circuit or brain region optogenetics, it is imperative that light penetrates the target area with sufficient irradiance to initiate retinoid activation. Optimal illumination should be conveyed to the target structure while inflicting minimal tissue damage. In behavioral studies involving animals, the stimulation protocol must also minimize interference with the animals' behaviors, necessitating lightweight implants and flexible tethers. The stimulation of entire circuits or regions is conventionally conducted using multimode optical fibers, which transport light from the source to the target area. A notable study in 2020 by Karl Deisseroth and his team involved positioning a 400 µm optical fiber above the intact skull surface, achieving light penetration to a depth of 7 mm and significant seizure suppression, demonstrating the potential for behavioral modulation without the need for surgical intervention.^[^ ^67^ ^]^ Subsequently, Jokubas Ausra et al. delivered high‐intensity optogenetic stimuli through the skull, facilitating not only miniaturized capacitive power storage but also enabling wireless optogenetics over an area exceeding 1m^2^ (**Figure** **4**a).^[^ ^68^ ^]^ However, high optical power may activate non‐target neurons or cause cellular damage. To enhance optogenetics efficiency, researchers like Jinghui Guo have employed transparent polystyrene microspheres as microlenses, utilizing the photon nano‐jet effect to focus incident light more precisely (Figure 4b).^[^ ^69^ ^]^ Recently developed was a self‐powered, rechargeable wireless optoelectronic system that can be fully implanted into animals, supporting continuous operation through non‐invasive wireless charging, critical for long‐term in vivo optogenetic studies and facilitating dynamic observation of neuromodulation over extended periods (Figure 4c).^[^ ^70^ ^]^ However, the behavioral area of freely moving animals is limited under rechargeable battery systems. Emerging spaceless optogenetic neuromodulation, based on solar power generation and capable of connecting to multiple devices via bluetooth, offers the significant advantage of enabling wireless charging without external instruments (Figure 4d).^[^ ^71^ ^]^ In a recent endeavor, a team led by Liping Zhou pioneered the development of a wireless, self‐powered optogenetic conditioning system (Figure 4e). This innovative device harnesses energy from human movement to produce the requisite optical illumination for optogenetic neuromodulation (ON).^[^ ^72^ ^]^ Notwithstanding, the application of fiber‐optic protocols presents significant obstacles in the modulation of dense, scattering tissues, often leading to tissue damage.^[^ ^73^ ^]^ An alternative approach utilizing upconversion nanomaterials enables the conversion of near‐infrared light (808 nm) to visible green light locally, obviating the need for optical fibers (Figure 4f).^[^ ^74^, ^75^, ^76^ ^]^ Illustratively, Yuqian Ma and colleagues have formulated an injectable ocular photoreceptor that integrates upconversion nanoparticles, facilitating infrared vision in murine model (Figure 4g).^[^ ^77^ ^]^ Despite these advancements, upconversion nanomaterials are not without limitations, such as the quantum efficiency of the upconversion process and potential adverse effects like brain heating.^[^ ^78^ ^]^ Sono‐optogenetics^[^ ^79^, ^80^ ^]^ represents a significant breakthrough in circumventing the limitations associated with fiber‐optic methods. This technique, leveraging the interplay between sound waves and optical mechanisms, enables high‐resolution neuromodulation via mechanoluminescent materials responsive to high‐frequency ultrasound.^[^ ^81^ ^]^ A noteworthy application of this method was demonstrated by Wenliang Wang and associates, who developed a liposomal nanolight source activated by focused ultrasound (FUS). This innovation permits temporal activation of neurons within both the superficial motor cortex and the deeper ventral tegmental area (VTA) following intracranial injection (Figure 4h).^[^ ^82^ ^]^

Until now optogenetics remains in the early clinical trial phase. The startup company Circuit Therapeutics, founded in 2010, plans to apply optogenetics to pain therapy. RetroSense Therapeutics officially initiated its optogenetics clinical trial for retinitis pigmentosa, the RST‐001 project, in 2016. They successfully completed the first patient's experiment by combining intraocular injection of an adeno‐associated virus (AAV) vector encoding ChrimsonR with light stimulation through engineered goggles.^[^ ^83^ ^]^ This marked the world's first clinical treatment using optogenetics. In 2020, the field of vision restoration showed particularly promising prospects, with two ongoing clinical trials (NCT02556736 and NCT03326336) utilizing optogenetics to treat retinal degeneration, a leading cause of blindness.^[^ ^84^, ^85^ ^]^ Given that optogenetic systems and exogenous cofactors are xenogeneic to humans, it is crucial to carefully evaluate and mitigate potential risks such as cytotoxicity and immune responses to ensure patient safety. Preliminary attempts have successfully used various optogenetic systems to modulate gene expression in rats, mice, and zebrafish, and there are already examples of applications in human stem cells.^[^ ^86^ ^]^

In optogenetics, one of the most important considerations for clinical applications is the use of unique promoters or specialized transfection methods.^[^ ^87^, ^88^ ^]^ The intensity of illumination and the duration of cell exposure are crucial factors affecting expression efficiency, as applied light irradiation can have side effects. Reducing invasiveness while improving light delivery efficiency are key considerations that currently limit in vivo applications of this technology. Solutions such as µLEDs, nanosphere microlenses, two‐photon microscopy, and upconversion nanomaterials are gradually enhancing optogenetic efficiency. Before clinical translation, it is imperative to address the safety of optogenetic gene expression concerning biocompatibility. Extensive preclinical testing is required to prevent immune responses and cytotoxicity caused by these exogenous protein molecules.

## 4 Thermogenetics

Thermal cues, characterized by their inherent reversibility, non‐invasive nature, deep tissue penetration, and ease of application, are ubiquitously present in biological contexts.^[^ ^35^, ^89^ ^]^ In recent years, a thermogenetic toolbox has emerged through the beneficial reuse of natural biological thermosensing mechanisms and through the construction of robust temperature‐sensitive receptors driven by innovative strategies inspired by synthetic biology.^[^ ^35^ ^]^ The amalgamation of this specific neuromodulation pathway with contemporary precise heat delivery technologies—encompassing magnetic thermotherapy, near‐infrared light, and focused ultrasound—facilitates wireless, precise, and deeply penetrating targeted neuromodulation in vivo. This chapter delineates recent progresses in thermogenetics‐based targeted neuromodulation over the preceding five years. It elucidates the technological merits and delineates the obstacles encountered in the translational journey towards clinical integration.

### 4.1 Genetically Engineered Thermal Switches for Cellular Activity

Temperature serves as a physical cue in nature that modulates the proliferation of a diverse array of microorganisms including archaea, bacteria, fungi, parasites, and viruses.^[^ ^90^ ^]^ The heat sensing are mediated by four principal classes of biomolecules: proteins, RNA thermometers (RNATs),^[^ ^91^ ^]^ DNA, and lipids. These intrinsic thermal sensing mechanisms offer a foundation for the development of sophisticated temperature sensors, which are integral components of the thermogenetic toolkit. Currently, the toolkit employs two primary methodologies: thermal induction and thermal inhibition, leveraging TS transcription factors and RNATs for the modulation of gene expression.^[^ ^35^ ^]^ Thermogenetics encompasses the activation of neurons through the stimulation of TRP cation channels or modifications in membrane capacitance. Nonetheless, the application scope of thermogenetics remains somewhat constrained due to a limited repertoire of suitable ion channels. A notable advancement in thermogenetic tools was the development of a neuronal activity inhibitor, derived from a temperature‐sensitive variant of the Drosophila Shibire protein, designated Shibirets1.^[^ ^92^ ^]^ This variant was engineered through a single amino acid substitution (G273D) within the Shibire protein, presenting a robust mechanism for cellular function disruption and facilitating the examination of neural circuits in Drosophila. R Subsequent innovations have introduced thermogenetic instruments based on the heat‐sensitive transient receptor potential (thermoTRP) channels.^[^ ^93^, ^94^ ^]^ Thermogenetics is limited by the TRP promoter and is much more sensitive. TRP channels exhibit conductivity that is three orders of magnitude greater than that of ChR (50–100 pS for TRP vs 40–60 fS for ChR2), thus ensuring a potent depolarizing impact even at minimal expression levels.^[^ ^95^, ^96^ ^]^ T The merits of thermogenetics extend beyond the high conductance of TRP channels, including superior tissue penetration capabilities and the absence of phototoxic effects.^[^ ^97^ ^]^ Moreover, the feasibility of applying thermal stimuli through environmental heating renders thermogenetics a minimally invasive and easily deployable technique.

### 4.2 Thermogenetic‐Mediated Precision Neuromodulation

**Figure 5:**
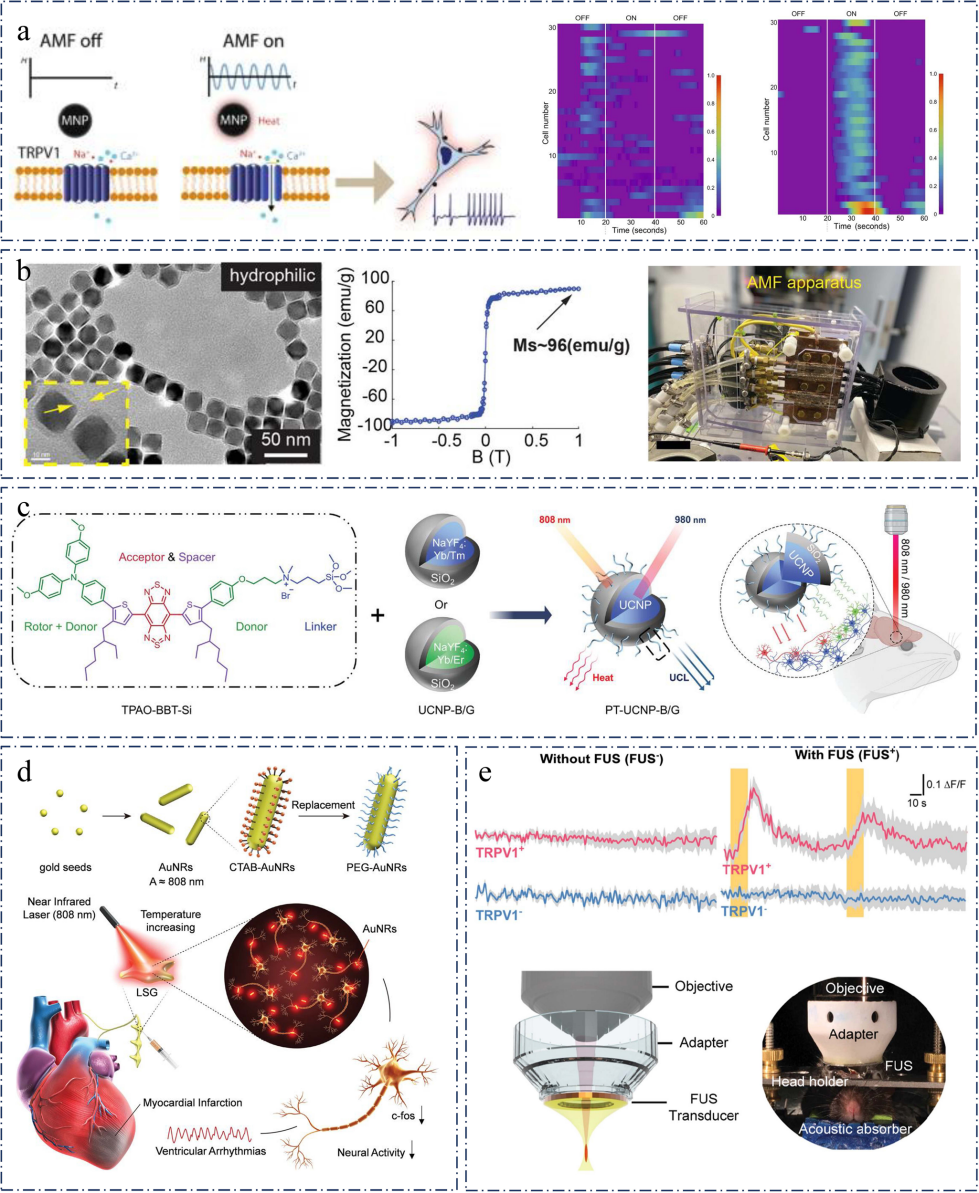
a) Left, magnetic field stimulation (“AMF ON”) induces magnetic nanoparticle heating and thereby membrane depolarization. Right, fluorescence increase was observed only in TRPV1+ cells with MNPs upon AMF application. Reproduced with permission.^[^ ^101^ ^]^ Copyright 2021, Springer Nature. b) Left, MNPs characterization. Middle, room temperature magnetization curve of MNPs and corresponding saturation magnetization (Ms). Right, magnetothermal stimulation of MNPs in magnetic apparatus. Scale bar: 5 cm. Reproduced with permission.^[^ ^103^ ^]^ Copyright 2022, Wiley‐VCH Verlag. c) Left, schematic illustration of the molecular design and the construction of PT–UCNP–B/G (two upconversion hybrid nanoparticles modified with photothermal agents). Right, schematic illustration of function and application of PT—UCNP–B/G in brain. Reproduced with permission.^[^ ^107^ ^]^ Copyright 2023, Wiley‐VCH Verlag GmbH. d) PEG–AuNRs photothermal neural inhibition. After the left thoracotomy and exposing the LSG, PEG–AuNRs were slowly injected into the LSG, and 808 nm NIR laser irradiation was performed vertically on the surface of the LSG. The resulting external NIR light–mediated PEG–AuNR photothermal heating suppressed the neural activity of the LSG and decreased the occurrence of VAs. Reproduced with permission.^[^ ^108^ ^]^ Copyright 2019, Wiley‐VCH Verlag. e) Above, average Ca^2+^ fluorescence intensity curves for mice with and without TRPV1 overexpression with FUS stimulation (FUS) and without FUS stimulation (FUS^+−^). Below, the custom‐designed system for simultaneous FUS stimulation and 2PM imaging. Reproduced with permission.^[^ ^110^ ^]^ Copyright 2021, Elsevier Inc.

The delivery of heat to neurological targets in a minimally invasive and safe manner is a crucial aspect of achieving targeted thermogenetic‐based neuromodulation, using the advanced thermoreceptors described above. The magneto‐thermal approach^[^ ^98^ ^]^ facilitates the generation of localized heating up to 42 °C in the presence of an alternating magnetic field,^[^ ^99^ ^]^ effectively activating heat‐sensitive ion channels, notably the transient receptor potential cation channel (TRPV1) thermosensory ion channels in the brain. Activation of these channels results in the opening of the TRPV1 channel, permitting the influx of calcium ions, and consequently inducing neural excitation, as illustrated in (Figure 5a).^[^ ^100^ ^]^ A noteworthy implementation of this technology is magnetothermal deep brain stimulation (mDBS), a wireless approach that activates deep brain circuits. This method involves the synthesis of magnetic nanoparticles targeting the TRPV1 receptor, which enables the activation of thermosensitive capsaicin receptors without the necessity for permanent hardware and connectors, showcasing significant improvements in motor deficits in Parkinson's disease models in both mild and severe cases (**Figure** **5**a).^[^ ^101^ ^]^ Further extending the application of magneto‐thermal techniques, the modulation of axonal growth has been utilized to foster neural regeneration. This approach employs magnetic nanoparticles (MNPs)^[^ ^102^ ^]^ and weak magnetic fields to remotely stimulate neural activity, offering novel avenues for enhancing neural recovery (Figure 5b).^[^ ^103^ ^]^ Additionally, the field of neuromodulation has explored optothermal effects, which can either stimulate or inhibit neuronal function. The primary agents in these processes are optothermal transducers, including plasmonic nanoparticles, silicon nanomaterials, and semiconducting polymer nanofixes.^[^ ^104^, ^105^, ^106^ ^]^ These transducers are classified based on their mechanism of action into three categories: 1) thermosensitive ion channels (e.g., TRPV1); 2) thermally driven changes in membrane capacitance; and 3) molecular thermotherapy strategies. Semiconducting polymer‐based optothermal nano‐transducers, in particular, have demonstrated the capacity for through‐scalp neuromodulation in freely moving animal models, underscoring the potential for non‐invasive therapeutic interventions (Figure 5c).^[^ ^107^ ^]^ Additionally, Ye et al. showed that Au nanorods can be used to inhibit LSG function and neural activity, thereby improving myocardial ischemia‐induced ventricular arrhythmias in a canine model (Figure 5d).^[^ ^108^ ^]^ Such findings illuminate the prospective utility of these nanorods in restoring the equilibrium within hyperactive neuronal circuits, presenting a novel therapeutic avenue for neurological disorders including epilepsy and Parkinson's disease. The employment of focused ultrasound (FUS) for thermal stimulation in deep‐seated tissues exemplifies another innovative approach, leveraging its excellent penetration capabilities. This methodology, termed sonothermogenetics,^[^ ^109^ ^]^ eschews traditional invasive techniques. Illustratively, a seminal study utilized FUS to precisely target the striatum deep within the brain, eliciting behavioral modifications in ambulatory murine models, as evidenced in (Figure 5e).^[^ ^110^ ^]^ This cell‐specific, non‐invasive strategy underscores the pivotal role of sonothermogenetics in advancing neural modulation therapies.

Thermogenetics has been successfully demonstrated in studies involving flies, zebrafish, and homeothermic animals such as mice. Magnetic nanoparticles (MNPs) have been optimized to dissipate heat effectively under clinically relevant alternating magnetic field (AMF) conditions, both in vitro and in freely moving wild‐type mice, as well as in mild and severe Parkinson's disease mouse models. Additionally, the potential of magnetothermal stimulation as a means to accelerate nerve growth suggests that MNPs could be delivered to injured nerves, with remote application of AMF enabling repeated activation of TRPV1 activity. Future research is expected to benefit from the in vivo application of this technology in injury models. Advanced Parkinson's disease patients often require the implantation of long‐term electrodes in deep brain regions like the globus pallidus and subthalamic nucleus to improve clinical conditions. Untethered deep brain stimulation offers new possibilities for treating neurological disorders; PT‐UCNP‐B can bidirectionally control disease symptoms without causing implant‐related injuries. The combination of PEG–AuNRs with NIR irradiation's photothermal effect represents a potentially valuable minimally invasive therapeutic strategy, allowing for remote manipulation of LSG neural activity with high spatiotemporal resolution, thereby improving ischemia‐induced VAs. Acoustothermal genetics uses viral constructs delivered to genetically selected neurons in mouse models, where wearable devices apply low‐intensity focused ultrasound to deliver small amounts of heat to selected neurons in the brain. This setup allows the ultrasound device worn on the head of freely moving mice to be moved to different locations across the brain, with potential future expansion to larger animals and possibly humans. In the future, unconventional forms of physical stimulation (such as mechanical forces, magnetic fields, and radio waves) could be used as heating tools in the development of thermogenetics, leveraging multimodal advantages for complementary integration.

Although thermogenetics has achieved significant breakthroughs, it still faces considerable limitations in therapeutic applications. First, the temperature range that mammals can tolerate (37–38 °C) is quite limited; temperatures reaching 43 °C can cause tissue damage.^[^ ^111^ ^]^ Most current research focuses on fruit flies and extending this technology to mammals presents several challenges. The rTRPV1 channel has been used for heat activation in cultured mammalian cells, but rTRPV1 typically activates at toxic high temperatures (> 42 °C),^[^ ^112^ ^]^ necessitating thermoTRP channels with more suitable characteristics. Genome mining for new thermoTRPs and mutagenesis of existing thermoTRPs can aid in addressing this need.^[^ ^111^ ^]^ Current research into deep‐tissue rapid localized infrared thermogenetics and FUS aims to address these issues. Recent advancements in in vivo optothermal neuromodulation have enabled tether‐ and implant‐free interfaces to modulate neural activity in deep brain regions of freely behaving animals with high temporal resolution.^[^ ^113^ ^]^ However, due to the inherent delays in system heating or cooling, this approach has relatively slow response times, making it more suitable for studying social interaction behaviors in animals.^[^ ^114^ ^]^ It is less effective than optogenetics for handling fast neurobiological events occurring on a millisecond timescale, especially when high temporal resolution is required for research and treatment of specific neural activities. Additionally, invasive intracranial injections may lead to acute invasiveness similar to other neuromodulation techniques.^[^ ^115^ ^]^ Moreover, during modulation, near‐infrared light can cause off‐target heating, potentially damaging nearby cells and tissues, leading to abnormalities in the structure and function of the nervous system.

## 5 Mechanogenetics

Cells within the human organism are perpetually subjected to mechanical stimuli, which are pivotal in orchestrating developmental processes, maintaining tissue equilibrium, and fostering regeneration. These forces also play a significant role in modulating various pathophysiological conditions.^[^ ^116^, ^117^ ^]^ Mechanical force receptors, integral to the cellular machinery, discern distinct patterns of mechanical stress, transducing these inputs into electrical or chemical signals that precipitate targeted biological responses. This transduction process opens avenues for refined neuromodulation through the manipulation of ion channel activities via specific mechanical stimulation protocols. Given their superior ability to penetrate deeper tissue layers and offer enhanced spatial and temporal resolution relative to photonic or thermal stimuli, mechanical forces are heralded as a forefront modality in precision electrical neurostimulation for clinical applications. This section elucidates on advanced mechanotransduction systems and explores the potential for precise deployment of mechanical stimuli in therapeutic settings.

### 5.1 Genetically Engineered Mechanical Switches for Cellular Activity

Mechanoreceptors constitute pore complexes that integrate and transduce mechanical stimuli into electrical or chemical signals through the insertion of specialized proteins.^[^ ^118^, ^119^, ^120^ ^]^ Entities serve as pivotal sensors within the nervous system, facilitating tactile and proprioceptive responses with rapid efficiency. The mechanotransduction process is mediated by various types of mechanosensitive (MS) channels, each characterized by distinct gating mechanisms.^[^ ^121^, ^122^ ^]^ Predominantly, in mammals, mechanotransduction involves two principal mechanisms: “force from lipid,” necessitating membrane conformational changes, and “force from tethering,” which depends on forces exerted by the extracellular matrix, the cytoskeleton, or both.^[^ ^123^ ^]^ Mechanosensory receptors encompass a heterogeneous group of entities, including, but not limited to, large conductance mechanoreceptors, piezoelectric mechanoreceptors, and transient receptor potential mechanoreceptors. These categories are extensively applied in the domain of mechanogenetics‐based neuromodulation, illustrating their significance in biomedical engineering. A landmark discovery by Sukharev et al. in 1994 identified two pivotal types of mechanosensitive channels in Escherichia coli: the large conductance mechanosensitive channel (MscL) and the small conductance mechanosensitive channel (MscS).^[^ ^124^, ^125^ ^]^ The MscL channels, comprising five identical subunits, are regulated by membrane tension and are notable for their significant conductivity and anion selectivity.^[^ ^126^ ^]^ Conversely, MscS channels are characterized by a lower activation threshold, enabling them to respond to minimal tensions.^[^ ^127^, ^128^, ^129^ ^]^ This intricate mechanistic understanding of mechanosensitive channels underscores the complexity and diversity of mechanotransduction processes, highlighting their crucial roles in sensory reception and signal transduction within biological systems. In 2010, Coste et al. elucidated the existence of a novel category of piezoelectric ion channels, denoted as Piezo.^[^ ^130^ ^]^ This discovery introduced two distinct members within this family: Piezo1 and Piezo2, each playing a critical role across various tissues and organs.^[^ ^130^, ^131^ ^]^ These channels are instrumental in sensing external mechanical stimuli and facilitating the operations of the autonomic nervous system.^[^ ^132^ ^]^ Piezo1 channels, predominantly located in non‐sensory organs such as the skin, lungs, kidneys, and bladder, stand out for their heightened sensitivity to mechanical forces, reacting to pressures as minimal as 10 pN.^[^ ^123^, ^130^, ^133^ ^]^ In contrast, Piezo2 channels,^[^ ^134^, ^135^ ^]^ which serve as the mammalian analogs to Piezo1, are predominantly situated in sensory‐rich tissues, including the trigeminal ganglion (TG),^[^ ^136^ ^]^ dorsal root ganglion (DRG) sensory neurons,^[^ ^137^ ^]^ and Merkel cells.^[^ ^138^, ^139^ ^]^ Furthermore, transient receptor potential(TRP) channels represent a diverse superfamily of non‐selective cation channels embedded within cell membranes, comprising tetrameric structures.^[^ ^140^, ^141^ ^]^ These channels are pivotal80 in mediating perceptions of pain, temperature, and taste.^[^ ^142^ ^]^ Based on amino acid sequences and 3D configureurations, have been systematically categorized into seven isoforms, spanning two classes.^[^ ^143^, ^144^ ^]^ The first class encompasses TRPC (TRP‐canonical), TRPV (TRP‐vanilloid), TRPM (TRP‐melastatin), TRPA (TRP‐ankyrin), and TRPN (TRPNompC). The second class includes TRPP (TRP‐polycystin) and TRPML (TRP‐mucolipin).^[^ ^145^, ^146^ ^]^ This classification underscores the functional diversity and complexity of TRP channels, highlighting their integral role in cellular signaling and sensory transduction processes.

### 5.2 Mechanogenetic‐Mediated Precision Neuromodulation

**Figure 6:**
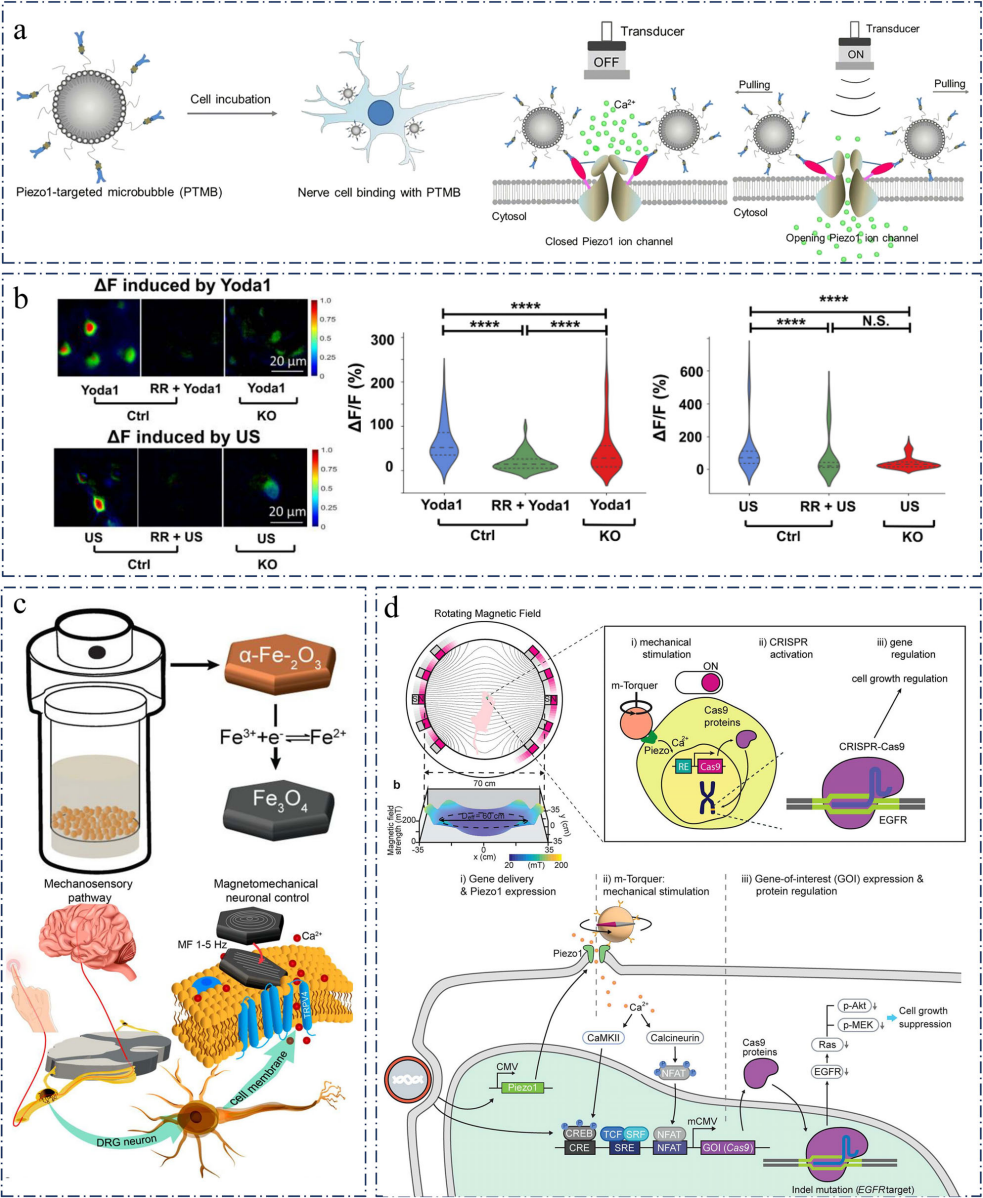
a) The diagram of PTMB (Piezo1‐targeted MBs) binding to the cells and the enhanced calcium influx by US stimulation. Reproduced with permission.^[^ ^152^ ^]^ Copyright 2021, Elsevier. b) Piezo1 contributes to neuronal responses to ultrasound stimuli in ex vivo brain slices. Left, representative images of ex vivo neuronal calcium signaling from brain slices of Ctrl or P1KO mice. Right, summarized calcium changes from ex vivo experiments. Reproduced with permission.^[^ ^154^ ^]^ Copyright 2023, National Academy of Sciences. c) Modulating mechanosensory cell activity through the transition from vortex to in‐plane magnetization magnetite nanodiscs. Reproduced with permission.^[^ ^156^ ^]^ Copyright 2020, American Chemical Society. d) Schematic diagram of the overall process of CRISPR. Reproduced with permission.^[^ ^157^ ^]^ Copyright 2022, American Chemical Society.

To attain precision in neuromodulation by activating mechanoreceptors, the application of mechanical loads that are efficient, controlled, and safe is essential. The primary mechanisms currently utilized for this purpose encompass ultrasound and magneto‐mechanical forces. Mechanogenetics approaches that utilize ultrasound exert mechanical force stimuli by subjecting cells or tissues to minute ultrasonic vibrations.^[^ ^147^ ^]^ This method effectively simulates the mechanical forces present in an organism's natural setting, thereby activating mechanoreceptors and initiating key cellular signaling pathways,^[^ ^148^, ^149^ ^]^ showcasing a wide array of biomedical applications.^[^ ^150^, ^151^ ^]^ In this context, a significant advancement was made by Xuelian Shen et al., who engineered a microbubble targeted at Piezo1 (PTMB) to amplify the effect of ultrasound on neuromodulation. Upon ultrasonic stimulation, the PTMB mechanism induces the opening of Piezo1 channels, leading to an influx of extracellular Ca^2+^ (**Figure** **6**a).^[^ ^152^ ^]^ This research illuminates the potential of ultrasound in neuromodulation applications. Furthering the investigation into Piezo1's involvement in ultrasound‐mediated neuromodulation, Jiejun Zhu et al. employed 85a Piezo1 conditional knockout (P1KO) mouse model to examine ultrasound‐induced neuronal activity both in vitro and in vivo. Their findings indicate that the influx of calcium induced by ultrasound was significantly reduced in neurons harvested from P1KO mice, and similarly diminished in neurons from normal mice pretreated with the Piezo1 inhibitor ruthenium red (RR) (Figure 6b)^[^ ^153^ ^]^, as compared to neurons exhibiting normal Piezo1 expression. These results affirm Piezo1's functional presence in the neurons of the mouse cerebral cortex and its integral role in the modulation of neuronal signaling by ultrasound in isolated brain slices.^[^ ^154^ ^]^

The seminal application of magneto‐mechanical forces within the ambit of mechanogenetics employs diminutive magnetic entities, termed magnetic particles. These entities, chiefly composed of magnetic iron oxide, demonstrate a pronounced sensitivity to external magnetic fields. Interaction of these particles with specified cells or tissues allows for the application of a magnetic stimulus. Upon the advent of an external magnetic field, magnetic particles are drawn to and localize within the cell or tissue, exerting a mechanical force. This methodology has been adeptly extrapolated to provide chronic stimulation to neuronal network models of fragile X syndrome (FXS),^[^ ^155^ ^]^ as delineated in the instance where Danijela Gregurec et al. engineered minuscule discs with nominal magnetic properties yet significant magnetic moments at low frequencies, namely a few Hertz. These discs facilitate the remote modulation of biological signals (Figure 6c).^[^ ^156^ ^]^ Consequentially, subsequent research activated mechanically sensitive ion channels, specifically Piezo1, on targeted cells via magnetic nanostructures to engender a magneto‐mechanical force. This force initiates the Ca^2+^ signaling pathway, thus effectively modulating intracellular proteins (Figure 6d).^[^ ^157^ ^]^ The innovative aspect of this magneto‐mechanically generated force technique is its transcendence of limitations related to the size of the organism or tissue depth, heralding its broad in vivo application potential in future endeavors.

Despite the significant potential of mechanically based genetic technologies in the field of neuromodulation, numerous challenges remain. The exact mechanisms of activation are not yet fully understood, as research indicates that various factors, including cavitation effects and acoustic radiation force, can lead to neuronal activation.^[^ ^158^, ^159^ ^]^ Mechanogenetics based on ultrasound must consider the complexity of signals and their impact range, particularly the trade‐off between penetration depth and spatial resolution—deeper penetration typically results in lower spatial resolution.^[^ ^160^ ^]^ Magnetomechanical neuromodulation is currently difficult to apply to freely moving large animals because this technology relies on large specialized equipment such as magnetic coils, limiting its flexibility and applicability.^[^ ^161^, ^162^ ^]^ To address the issue of penetration depth, the application of nanotransducers becomes crucial. Additionally, both ultrasound and radio frequency technologies encounter potential interference between recording and stimulation signals in practical applications. However, this challenge can be effectively mitigated through multiplexing methods in both the time and frequency domains.^[^ ^163^ ^]^ The temporal resolution of mechanogenetics remains a key focus for future research.^[^ ^164^ ^]^ As technology advances, addressing these issues will facilitate further development in the field, enabling it to play a more significant role in both clinical and basic research.

## 6 Chemogenetics

Chemogenetics represents a burgeoning field that enables the modulation of neural activity with unprecedented precision.^[^ ^165^, ^166^, ^167^ ^]^ This technique involves the genetic engineering of exogenous ligands to selectively bind to specific receptors, thus offering a targeted approach to neural modulation.^[^ ^168^, ^169^ ^]^ The foundational technology primarily utilizes G protein‐coupled receptors (GPCRs) engineered to respond exclusively to bespoke ligands—designer receptors exclusively activated by designer drugs (DREADDs).^[^ ^170^, ^171^ ^]^ This chemogenetic approach surpasses traditional pharmacological methods^[^ ^172^, ^173^ ^]^ by leveraging biologically inert ligands, such as clozapine N‐oxide (CNO),^[^ ^174^ ^]^ and receptors genetically modified to be activated solely by these specific ligands, thereby eliminating cross‐reactivity with endogenous neurotransmitters.^[^ ^175^ ^]^ Currently, 30% of approved drugs have a G protein‐coupled receptor (GPCR) as their primary target.^[^ ^176^, ^177^, ^178^ ^]^ Additionally, ligand‐gated ion channels (LGICs) are also commonly targeted in chemogenetics.^[^ ^172^, ^179^ ^]^ The type of G protein coupled to the receptor determines the cell signaling cascade response.^[^ ^180^ ^]^ Initially, muscarinic acetylcholine receptors (mAChRs) served as the template for DREADD development,^[^ ^181^ ^]^ with variants such as hM3Dq and hM4Di being engineered to interface with Gq and Gi proteins, respectively.^[^ ^171^, ^182^ ^]^ An alternative strategy involved kappa opioid receptors (KORs),^[^ ^183^, ^184^ ^]^ another GPCR category, which were modified to be activated by salvinorin B and coupled to Gi proteins for bidirectional neural regulation.^[^ ^182^, ^185^ ^]^ Unlike the indirect modulation achieved through GPCR‐based chemogenetics, LGICs enable direct control over neuronal electrical activity via gene regulation.^[^ ^186^, ^187^ ^]^ Despite the potential of chemogenetics, the field has encountered obstacles related to the limitations of small‐molecule agonists in developing effective tools. To surmount these challenges, Sternson and colleagues introduced a drug‐selective agonist module (PSAM). The PSAM reduces its affinity for endogenous acetylcholine and binds to the ion pore of either the excitatory cation‐selective serotonin receptor 3 (5‐HT3) or the inhibitory chloride‐selective glycine receptor structural domains. This leads to neuronal activation or silencing of PSEM agonists, respectively.^[^ ^188^ ^]^

**Figure 7:**
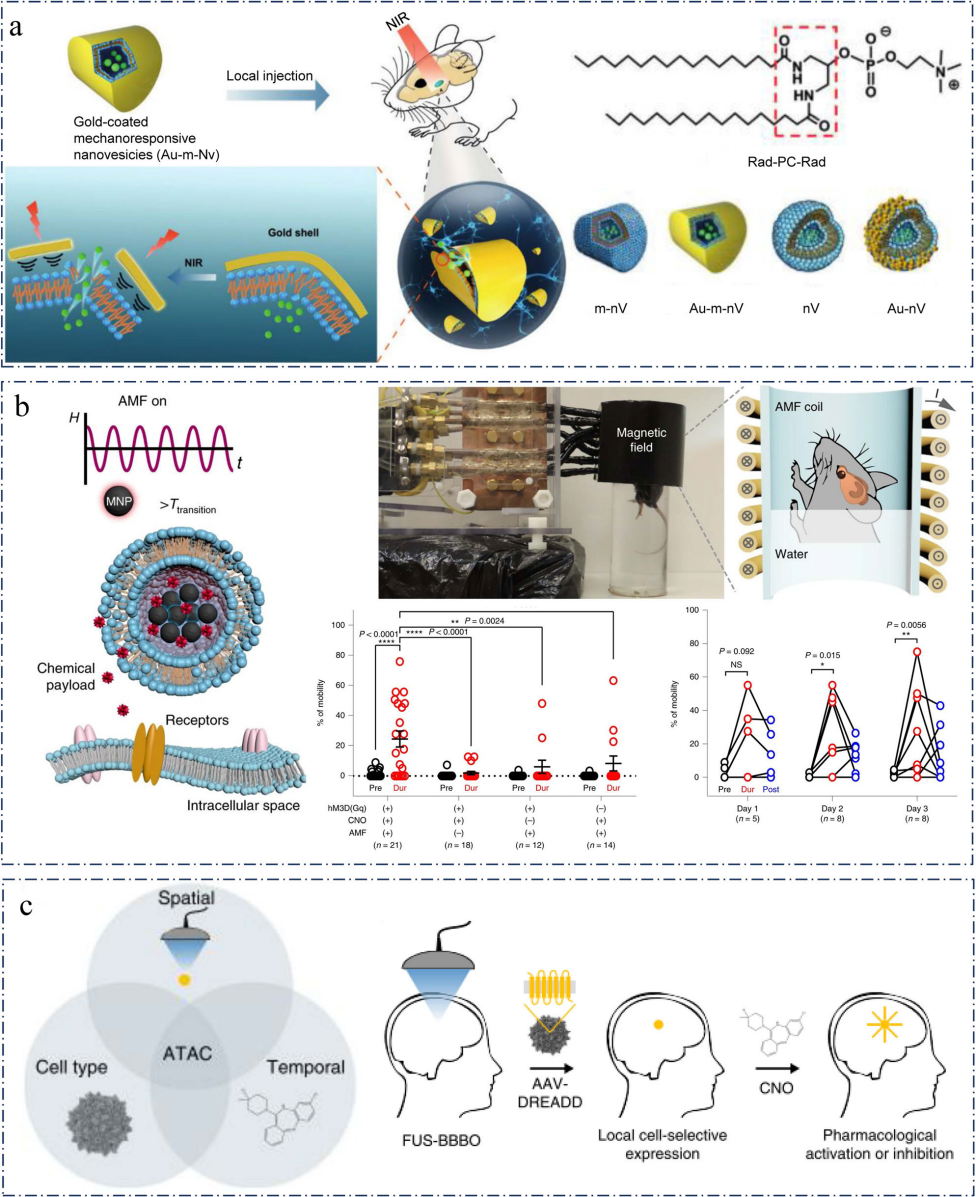
a) Schematic illustration of near‐infrared (NIR) laser pulsetriggered release in deep brain regions using gold‐coated mechanoresponsive nanovesicles (Au‐m‐nV). Reproduced with permission.^[^ ^190^ ^]^ Copyright 2020, Wiley. b) Left, experimental scheme of the AMF‐triggered chemical payload release from the magnetoliposomes. Right, Remote chemomagnetic modulation of mouse behaviour using chemogenetics. Reproduced with permission.^[^ ^191^ ^]^ Copyright 2019, Springer Nature. c) The ATAC (acoustically targeted chemogenetics paradigm) provides a combination of millimetre‐precision spatial targeting using FUS, cellular specificity using viral vectors with cell‐type‐specific promoters driving the expression of chemogenetic receptors, and temporal control through administration of the chemogenetic ligand. Reproduced with permission.^[^ ^192^ ^]^ Copyright 2018, Springer Nature.

Chemical neuromodulation typically involves three neuromodulators,^[^ ^189^ ^]^ the first of which can be released by light. In the realm of chemical neuromodulation, innovative strategies such as the ultrarapid nanotransducer exemplify the application of nanotechnology in neuroscience. This method employs gold‐coated, mechanically responsive nanovesicles for the precise delivery of bioactive molecules into brain tissue, enabling the modulation of neural activity in deep brain regions, as demonstrated by the targeted release of calcineurin in a study depicted in **Figure** **7**a.^[^ ^190^ ^]^ The utilization of magnetic fields for the remote heating of nanotransducers constitutes a novel approach for the controlled release of encapsulated molecules. Rao et al. pioneered a chemical‐magnetic methodology to stimulate endogenous circuits through the regulated liberation of receptor ligands. Under an alternating magnetic field (AMF), magnetic nanoparticles induce thermal responses, leading to the expeditious release (within 20 sec latency) of molecules from thermally responsive lipid vesicles, facilitating a non‐invasive mechanism for the modulation of neural circuits as illustrated in Figure 7b.^[^ ^191^ ^]^ Moreover, conventional strategies for the systemic administration of exogenous ligand‐based drugs are fraught with potential complications, including adverse reactions, immune responses, and tissue toxicity. The acoustic‐targeted chemogenetics (ATAC) strategy emerges as a solution to these challenges.^[^ ^192^ ^]^ This method employs microbubble‐enhanced FUS to breach the blood‐brain barrier^[^ ^189^ ^]^ utilizes adeno‐associated viral (AAV) vectors for the precise delivery of genes to specific cells.^[^ ^193^ ^]^ This approach incorporates the use of designer receptors exclusively activated by designer drugs (DREADDs), depicted in Figure 7c, to ensure targeted and efficient neuromodulation.

Recently, chemogenetics has made considerable progress in diseases such as obesity, sleep disorders, and epilepsy.^[^ ^194^, ^195^, ^196^ ^]^ The effectiveness of chemogenetics in locally reducing hippocampal seizures has been demonstrated. Recently developed techniques enable repeatable and minimally invasive perturbations of almost identical neuronal populations in the brains of large primates, combined with whole‐brain functional imaging and neuronal recording, providing valuable opportunities for causal studies on the function of frontotemporal networks in object memory.^[^ ^197^ ^]^ Simultaneously, chemogenetic inhibition of infrapyramidal neurons has shown potent antiepileptogenic effects.^[^ ^198^ ^]^ Compared to traditional optogenetic fiber‐optic invasion, Sawada et al.^[^ ^194^ ^]^ designed a chemogenetic tool–SYNCit–that can regulate synaptic strength by systematically administering an inert chemical dimer without fiber‐optic invasion,^[^ ^199^ ^]^ thereby facilitating its application in homeostatic sleep regulation. Subcutaneous injection of clozapine‐N‐oxide or declozaxamine in rats to activate LH–hM4D (Gi) effectively reduced food intake. The current potential of chemogenetic receptors as precise neural modulation methods, where the mechanism of action is directly derived from molecular pathophysiology rather than rationalized based on neuronal excitability, remains largely conceptual.

The translational therapeutic potential of chemogenetics primarily depends on the ability to express chemogenetic receptors in specific cell types. Effective neuromodulation requires precise spatial, cell‐type, and projection‐specific identification and targeting of discrete cells to achieve the desired outcomes and avoid off‐target effects.^[^ ^167^ ^]^ First, appropriate delivery methods for recombinant adeno‐associated virus vectors (rAAVs) are crucial to minimizing off‐target effects.^[^ ^200^ ^]^ Programmable, cell‐type specific transgene expression must be established.^[^ ^167^ ^]^ Additionally, therapeutic applications of chemogenetics must establish specific receptor‐ligand interactions and minimize off‐target effects. The dose thresholds for DREADD activation depend on the cellular and molecular characteristics of the target brain regions.^[^ ^201^, ^202^ ^]^ Future research must rigorously evaluate the safety and pharmacological properties of ligands to ensure their suitability for therapeutic use.

## 7 Electrogenetics

**Figure 8:**
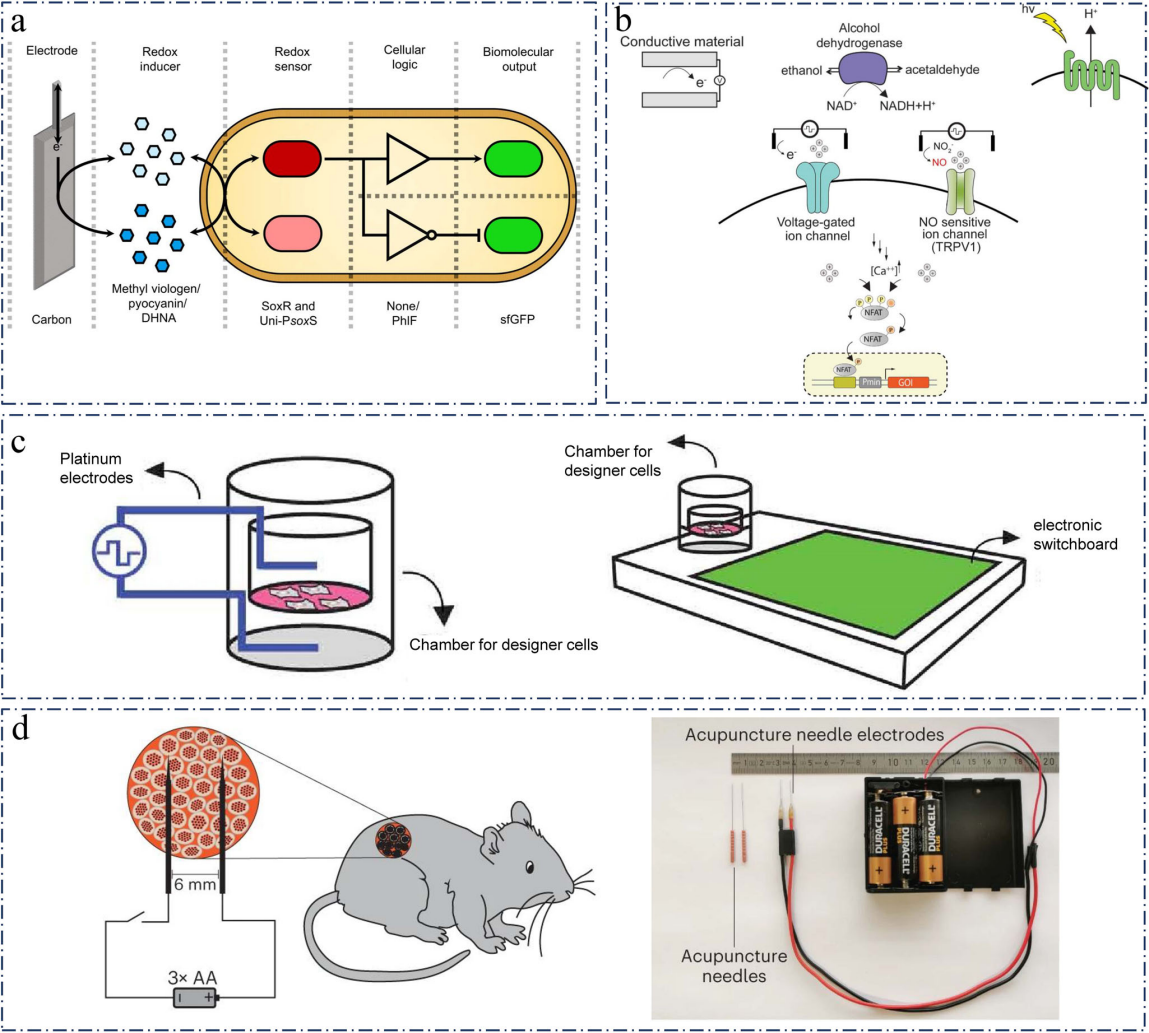
a) Electrogenetic systems. Reproduced with permission.^[^ ^213^ ^]^ Copyright 2022, AMER ASSOC ADVANCEMENT SCIENCE. b) Tools and strategies for generation of electro‐sensitive designer cells. Reproduced with permission.^[^ ^216^ ^]^ Copyright 2022, Elsevier. c) Bioelectronic implant in vitro. Reproduced with permission.^[^ ^216^ ^]^ Copyright 2022, ELSEVIER SCI LTD. d) Scheme showing how encapsulated DART‐engineered cells implanted in the back of mice are stimulated and the customized acupuncture needles connected to the alkaline batteries. Reproduced with permission.^[^ ^217^ ^]^ Copyright 2023, Springer Nature.

Electrical stimulation has long been employed in clinical practices, including deep brain,^[^ ^203^ ^]^ vagus nerve,^[^ ^204^ ^]^ and spinal cord electrical stimulation,^[^ ^205^ ^]^ to modulate the activity of excitable nerve cells. Traditional methods of direct electrical stimulation, however, lack the specificity for precise neuromodulation, affecting all cells within the electrical field at the target site indiscriminately. Recent advancements in electrogenetics have introduced a paradigm shift, enabling precise control over the activity of genetically engineered cells using electrical fields. This approach has overcome the limitations of direct electrical stimulation, promising targeted neuromodulation.^[^ ^206^ ^]^ The application of relatively simple, inexpensive, and widely accessible electronic devices for direct, cofactor‐free radiofrequency stimulation of engineered cells represents a significant advancement. Utilizing micrometer‐sized electrodes, this method allows for robust, tunable, and reproducible stimulation. Furthermore, it facilitates the development of devices that are more compact and miniaturized than those necessitated by other physical stimuli, such as light, heat, or magnetic fields.^[^ ^207^, ^208^, ^209^ ^]^ Electrogenetics leverages the generation of electric fields to facilitate the transfer of free electrons, enabling the targeted activation of engineered cells. This activation encompasses both direct and indirect method (**Figure** **8**b). Direct activation involves the electric field inducing a conformational change in the receptor platform of the engineered cell, exemplified by the action of voltage‐gated ion channels in neurons, which regulate membrane polarization through the fast conduction of ions.^[^ ^210^ ^]^ Indirect activation strategies, conversely utilize cells engineered to sense the byproducts of electrical stimulation, rather than the electric field itself. An example of this is the electrochemical control of gene expression, illustrating the integration of electronic and genetic circuits. These methodologies, as highlighted in Figure 8b, underscore the versatility and potential of electrogenetics in precise neuromodulation.^[^ ^211^, ^212^ ^]^ The electrogenetic system is composed of four key components: the electric level, the redox inducer, the redox transcription factors, and their cognate promoters (Figure 8a).^[^ ^213^ ^]^ Redox modelling acts as a mechanism to link electrons with biological systems^[^ ^212^, ^214^ ^]^ thereby enabling the regulation of gene expression through alterations in the electrical potential level. Tschirhart et al. utilized exogenous redox mediators in E. coli to achieve electronic control over synthetic genetic networks.^[^ ^215^ ^]^ Following this, Krawczyk et al. applied radiofrequency stimulation to electrobacterial cells within a specialized bioelectronic device to manage blood glucose levels in mice with type 1 diabetes (Figure 8c).^[^ ^44^, ^216^ ^]^ A subsequent development allowed for wearable electronic devices to be programmed directly for therapeutic use (Figure 8d).^[^ ^217^ ^]^

Furthermore, the integration of advanced radio‐stimulation protocols with electrogenetics offers a pathway toward less invasive methods of neuromodulation. This includes the use of ultrasound–electric conversion technology and magneto–electric technology. In 2012, a method was proposed for non‐invasive brain stimulation in patients with Parkinson's disease, which converts an external magnetic field into a local electric field to modulate cellular activity.^[^ ^218^ ^]^ In 2022, researchers developed a self‐powered, self‐controlled voltage‐driven system based on piezoelectric materials for programming inductive design units.^[^ ^219^ ^]^ For clinical translation, verifying the lifespan of these piezoelectric devices is crucial. Experimental results showed that all piezoelectric implants exhibited high biocompatibility throughout the 30 day study period and provided a reliable power source for electrically triggered bio‐drug release.^[^ ^220^ ^]^ In clinical applications, this device can significantly simplify the treatment regimen for patients with type 1 diabetes who rely on frequent insulin injections, potentially improving patient adherence. Moreover, this technological advancement heralds significant potential for electrogene therapy in next‐generation cell therapies, offering new hope for treating other diseases.

However, the clinical translation of electrogene therapy still faces several challenges. First, introducing exogenous electroactive molecules or regulatory genes into the body may cause cytotoxicity, inflammation, and other safety issues, posing potential risks for clinical application. Second, research on deeper mechanisms of action remains limited. The specific ways in which electroactive molecules or regulatory genes function and interact with cellular signaling pathways require further investigation and exploration. Most studies are currently at the proof‐of‐concept stage, with a considerable distance to practical application. Additionally, the field of electrogene therapy lacks effective and standardized tools and technologies. The required equipment and experimental conditions are complex, presenting significant obstacles to both research and clinical application. Apart from preliminary explorations in diabetes treatment, electrogene therapy has seen relatively little research in treating other diseases in recent years, limiting its broader application potential.

## 8 Magnetogenetics

External direct current (DC) and alternating current (AC) magnetic fields easily penetrate biological tissues, can be readily generated by current‐carrying wires or permanent magnets,^[^ ^221^ ^]^ and can activate any neuronal population regardless of its anatomical location without invasive surgery. Ideally, one could leverage the sensitivity of nature's magnetoreceptors to achieve this goal. The characteristics of magnetic fields are commonly utilized in medical diagnostic applications, such as magnetic resonance imaging (MRI),^[^ ^222^ ^]^ and there is strong motivation to apply the same advantages of magnetic fields to control biological functions, akin to transcranial magnetic stimulation (TMS).^[^ ^223^ ^]^ This chapter reviews non‐invasive genetic approaches that combine magnetoreceptors with remote magnetic stimulation.

**Figure 9:**
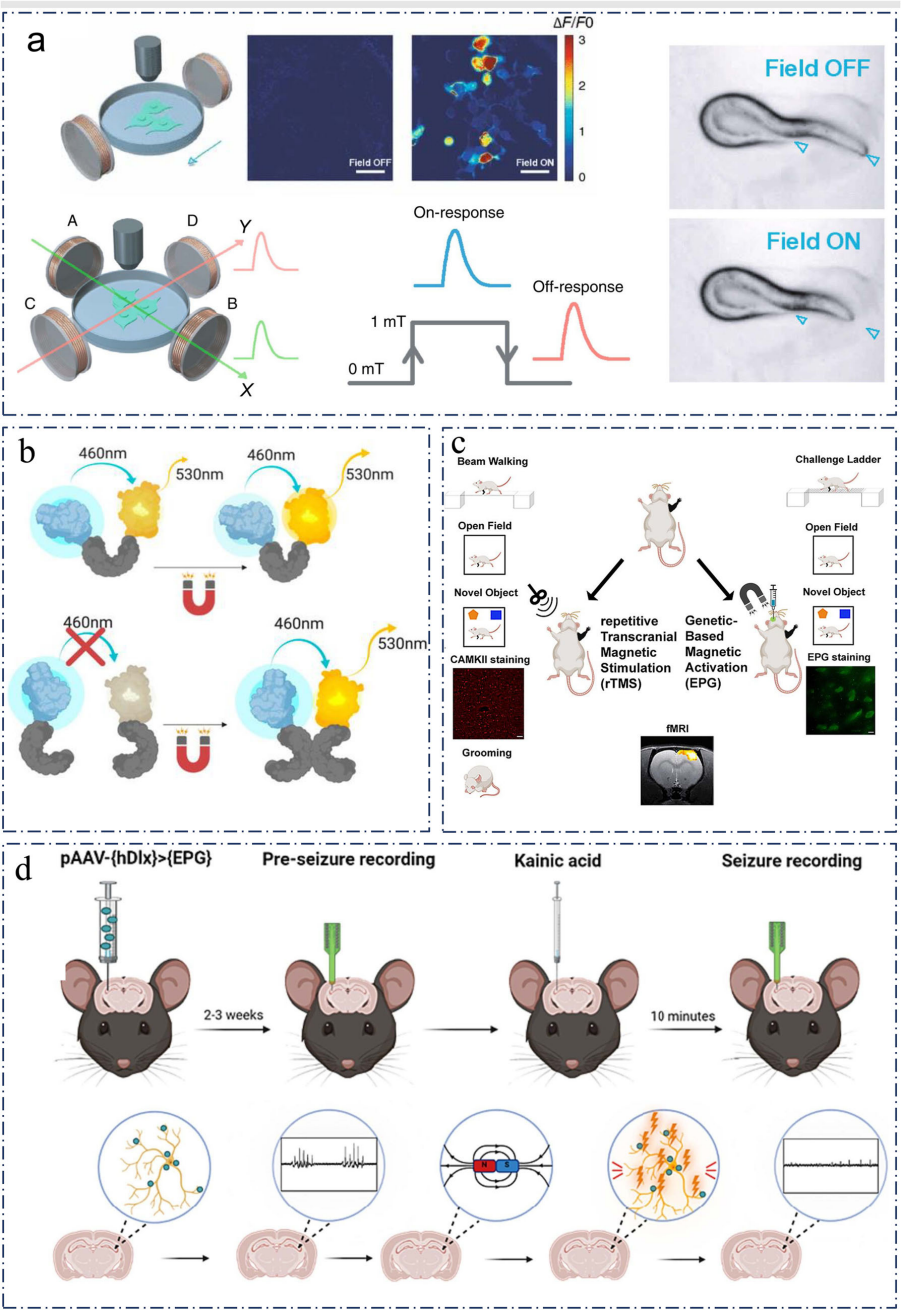
a) Schematic of magnetic stimulation of MAR‐GCaMP6s co‐transfected HEK‐293 cells by a pair of electrical coils. On‐response and off‐response patterns of neuronal activity. Schematic showing that switch‐on and switch‐off of magnetic field induced on‐response (blue trace) and off‐response (red trace) patterns of neuronal activity. Reproduced with permission.^[^ ^246^ ^]^ Copyright 2015, Elsevier. b) Bioluminescent resonance energy transfer studies of EPG conformational changes. Reproduced with permission.^[^ ^43^ ^]^ Copyright 2024, Frontiers. c) Non‐invasive neuromodulation using rTMS and the electromagnetic‐perceptive gene (EPG) facilitates plasticity after nerve injury. Reproduced with permission.^[^ ^227^ ^]^ Copyright 2020, Elsevier. d) Potential mechanism of action: EPG is activated in response to magnetic fields generated by neuronal firing during seizures in the hippocampus. Reproduced with permission.^[^ ^230^ ^]^ Copyright 2023, Elsevier.

Two main theories explain the conversion of magnetic information into neuronal stimulation. The first is the magnetite theory, which posits that animals possess an intracellular compass composed of magnetite (Fe_3_O_4_) crystals coupled to mechanosensitive ion channels.^[^ ^224^ ^]^ According to changes in the local magnetic field's strength and/or polarity, these crystals exert forces on channel proteins, leading to calcium ion influx into cells. However, there is a lack of direct evidence supporting its efficacy. Currently, there are three primary types of artificial receptors: the first uses radiofrequency (RF) waves to activate transient receptor potential channels (TRPV1 and TRPV4) coupled with cellular ferritin;^[^ ^225^ ^]^ the second involves nanoparticles that respond to force or torque; and the third is the iron‐chaperone protein ISCA1.^[^ ^224^ ^]^ The first two methods essentially achieve neural modulation through heat effects or mechanical forces induced by magnetic fields, as reviewed in thermogenetics and mechanogenetics. For the last type, ISCA1, it binds a small number of iron atoms and is associated with human mitochondrial dysfunction syndromes, universally expressed in all cell types of eukaryotes. Long et al. demonstrated that applying external magnetic fields to HEK‐293 cells and cultured hippocampal neurons expressing this magnetoreceptor led to repeatable and reversible membrane depolarization and calcium influx (**Figure** **9**a). EPG is a magnetosensitive, membrane‐associated protein discovered in recent years.^[^ ^226^, ^227^, ^228^, ^229^ ^]^ This magnetic sensor undergoes conformational changes under the influence of magnetic stimulation (Figure 9b). Cywiak et al.^[^ ^227^ ^]^ developed a novel minimally invasive wireless technology based on EPG and found that non‐invasive neural modulation using repetitive transcranial magnetic stimulation (rTMS) and electromagnetic‐sensing genes (EPG) promotes plasticity following neural injury (Figure 9c). This represents the first application of EPG intervention in animal models of injury. Metto et al.^[^ ^230^ ^]^ developed a new closed‐loop method using magnetogenetics to control seizure activity by encoding proteins that respond to magnetic fields (Figure 9d). Expression of EPG in inhibitory hippocampal neurons can provide on‐demand suppression of seizures, where EPG simultaneously acts as a seizure detection component and neuronal regulator, offering an on‐demand closed‐loop system. Specifically, while TMS can activate a small region of the cerebral cortex, its control over specific neuronal types is limited. In contrast, magnetogenetics, utilizing cell‐type specific promoters, can express magnetosensitive proteins in specific cell types, enabling more precise neural modulation. This approach has the potential to significantly improve treatments for Parkinson's disease and other neurological and neuropsychiatric disorders.

It is worth emphasizing that the current incarnation of magnetogenetics faces several significant challenges. First, it remains unclear how particles dependent on genetically encoded ferritin nanoparticles actually work. Second, the equipment required for magnetic fields is costly, and generating powerful magnetic fields poses a risk of introducing artifacts. Finally, its effectiveness is not yet ideal, with neuron activation being much slower compared to optogenetics. Developing robust and reliable magnetogenetic sensors is no easy task.

## 9 Conclusions and Outlook

Genetic‐based targeted neuromodulation represents a pivotal tool in molecular biology and biomedical engineering, facilitating precise modulation of distinct neuronal populations and the dynamics of neural circuits. Recent advancements, particularly in the last five years have significantly contributed to the understanding of cellular biological processes, neural circuit mapping, and the management of neurological and psychiatric disorders resistant to pharmacological treatments. The integration of wirelessly operated driving fields and the use of implantable micro‐ and nanoscale transducers in vitro mark a departure from traditional device designs that were initially based on cardiac pacemakers in the 1950s. This innovation improves spatial resolution, patient compliance, and biosafety in neuromodulation techniques. Despite these advancements, broader clinical adoption and commercialization face challenges, notably:

1. Safety considerations: The application of genetic strategies involves the introduction of endogenous or exogenous genes and the sustained expression of genetic modulators for neuromodulation purposes. Ensuring the safety of these genetic applications for neurons and surrounding tissues is imperative, as some genetic modulators may induce cytotoxicity, inflammatory responses, or irreversible damage. Therefore, biomarker detection techniques and imaging techniques such as in vivo imaging and fluorescence microscopy are used to monitor the effects of the application of genetic tools in real time and to manage any adverse reactions. Meanwhile, the selection of nanocarriers with high stability can mitigate cytotoxicity. Therefore, biomarker detection technologies and imaging techniques such as in vivo imaging and fluorescence microscopy are used to monitor treatment effects. Unlike traditional methods, nanoprobes^[^ ^231^, ^232^ ^]^ are being constructed to assist with biomarker detection, serve as gene carriers, and provide closed‐loop feedback control, thereby enabling real‐time monitoring and management of any adverse reactions. Additionally, selecting biomaterials with high stability and good biocompatibility can reduce their residual amount and duration in the body, offering an alternative approach.^[^ ^233^, ^234^ ^]^ Recently, there has been increasing attention on novel functionalized and specially designed nanomaterials. Moreover, the long‐term toxic effects of nanomaterials and their potential impacts on normal cells await future solutions.
2. Ligand‐receptor considerations: For the issues of ligand‐receptor and applicability differences, researchers need to seriously consider and resolve problems related to ligand and receptor selection, specificity, signaling pathways, time windows, tolerance, and drug resistance.^[^ ^235^ ^]^ To optimize the selection of ligands and receptors, personalized design and adaptive ligand applications are critical.^[^ ^236^, ^237^ ^]^ By developing multifunctional receptors and intelligent switch detection systems, more precise targeted therapies can be achieved.^[^ ^238^, ^239^ ^]^ Furthermore, optimizing receptor structures using gene editing and protein engineering technologies can finely regulate signaling pathways, enhancing therapeutic effects while reducing side effects. To address drug resistance and tolerance, real‐time monitoring and combination therapies are essential. Employing biomarker detection technologies and in vivo imaging techniques (such as fluorescence microscopy) allows for real‐time monitoring of treatment efficacy and swift handling of any adverse reactions. Unlike traditional methods, constructing nanoprobes can not only assist in detecting biomarkers and gene carriers but also achieve closed‐loop feedback control, ensuring dynamic adjustments during therapy. Selecting materials with high stability and good biocompatibility can reduce in vivo retention time and potential toxicity, further enhancing safety. Recently, novel functionalized and specially structured nanomaterials have received increasing attention, but their long‐term toxicity and impact on normal cells still require in‐depth research.
3. Scalability, cost control, and standardization: Biological genetic technologies face multiple challenges. First, the efficiency and versatility of delivery systems are critical bottlenecks when achieving large‐scale applications. Commonly used viral vectors, such as rAAV,^[^ ^240^ ^]^ are highly effective but have limited production capacity and are expensive, restricting their widespread use. To address the high costs associated with advanced gene delivery systems, it is essential to develop more economical and efficient carrier systems and reduce raw material consumption. Moreover, significant differences in equipment and technology platforms across different laboratories and medical institutions make it difficult to ensure consistency and reproducibility. The large‐scale deployment capability of these technologies urgently needs improvement. Adopting automated equipment and standardized operating procedures (SOPs) can achieve efficient and stable gene editing and delivery processes.
4. Ethical considerations: The application of gene regulation technology in humans involves complex ethical issues, including informed consent, privacy protection, and the risk of potential misuse, all of which must be handled with caution. Given that gene editing can introduce off‐target gene expression or unintended mutations, long‐term risks require ongoing monitoring and research. To ensure safety and ethical integrity, all gene editing projects should undergo rigorous ethical review, adhere to internationally recognized ethical principles, and maintain transparency by promptly communicating updates to the public. Governments and international organizations should collaborate to establish unified standards and guidelines that guide the responsible development of this technology along with oversight mechanisms to ensure compliance. Special emphasis is placed on obtaining informed consent, ensuring participants fully understand the risks and benefits, and have the right to withdraw at any time. For vulnerable groups, such as minors or individuals with cognitive impairments, additional safeguards are recommended, like joint decision‐making with guardians. Furthermore, we advocate for promoting public participation and social dialogue through various platforms to enhance societal understanding and support for the technology, thereby reducing misunderstandings and concerns.

Despite the challenges in clinical application, genetic‐based targeted neuromodulation holds significant promise in biomedical engineering. Interdisciplinary collaboration is key to developing safer and more effective targeting and implementation protocols, aiming to make clinical neuromodulation a standard practice. Looking ahead, the field aims for:

1. Individualized treatment: The potential for precise, personalized therapeutic approaches through genetic neuromodulation technology is significant. By leveraging advancements in heat‐sensitive molecules, gene editing, and targeted delivery systems, it is possible to achieve high precision in targeting specific neuron types or circuits. This enables the development of personalized treatment plans tailored to the patient's specific genetic makeup, disease characteristics, and treatment responses, thereby improving treatment efficacy and safety while reducing side effects.
2. Treatment of drug‐refractory neurological disorders: Advanced genetic neuromodulation methods present novel avenues for the treatment of neurological disorders. These methodologies are capable of normalizing aberrant neuronal activities or mending impaired neural pathways, thereby ameliorating symptoms and functional deficits tied to a range of neurological ailments. Furthermore, they furnish innovative approaches for the rehabilitation of nerve injuries and neurodegenerative conditions. Through the promotion of nerve regeneration, enhancement of neuronal functionality, or the reconstitution of damaged neural networks, the objective is to reinstate the functionality of the compromised regions.
3. Cerebral analysis and cognitive studies: Neuromodulation strategies in genetics play a pivotal role in advancing our understanding of the cerebral architecture and its functionalities. The precise identification, manipulation, and observation of specific neurons or neural circuits can unravel the underlying principles governing brain activities, encompassing cognitive operations, as well as learning and memory dynamics. Such insights are instrumental in decoding the interplay and regulatory mechanisms among the constituents of each neural consortium, thereby paving the way for novel therapeutic interventions for related pathologies.
4. Neuro‐computer interfaces and human‐computer interaction: The amalgamation of genetic neuromodulation and neuro‐computer interface technologies holds the promise of facilitating direct, wireless communication and interaction between the human brain and external apparatus. This convergence heralds unprecedented prospects, such as the cerebral control of external devices and the augmentation of human cognitive capabilities.
5. Precision in genetics‐based instrumentation: The deployment of genetics‐based apparatus necessitates the integration of intricate electronic devices, advanced artificial intelligence‐supported software, and an external power source for operational control. It is imperative to judiciously harness the capabilities of extant, sophisticated intelligent systems to foster cross‐disciplinary collaboration, thereby furthering the innovation of new‐age, digitally precise diagnostic and therapeutic tools. Such advancements are crucial for the realization of convenient, precise, and manipulable diagnostics and therapeutic interventions. In today's digital era, the capacity for remote, off‐site program management has become a fundamental component of neuromodulation technology evolution, particularly within tertiary healthcare facilities worldwide. The rapid progression of neuromodulation technology, propelled by the societal challenges of an aging population, breakthroughs in neuroscience, and the evolution of accompanying engineering solutions, necessitates continued clinical and mechanistic investigations to broaden its applicability and enhance therapeutic efficacy.

It is worth stressing again that genetics‐based technologies are largely still in the laboratory research or early clinical trial stages and have not yet reached a level of maturity for widespread application or commercialization. This review focuses on the current research progress of genetics‐based precision neural modulation technologies and discusses key areas for future research, including technology optimization, safety assessments, and pathways for clinical translation, to ensure these technologies can be safely and effectively applied in clinical practice. Despite several challenges that remain, transitioning to clinical application will require extensive long‐term exploration and research in in vivo animal models. However, it is foreseeable that with deeper research and technological advancements, genetics‐based technologies are undergoing rapid development and exhibit significant potential for research and application.

## 10 Genetics‐based artificial artefacts in neuromodulation processes

In the practical application of genetics‐based neural modulation technologies, experimental results can be influenced by multiple artifacts that reduce data accuracy and reliability. To help beginners better understand and address these issues, this section summarizes common artifact types and their avoidance strategies.

1. Light scattering can lead to non‐specific activation or inhibition of neurons and excessive light intensity may cause phototoxic effects, impacting cell function, and potentially leading to neuronal death. We recommend using the minimum effective light intensity and selecting specific wavelengths to minimize effects on non‐target cells.
2. Variations in temperature can alter the physiological activity of target cells, particularly in the expression of heat shock proteins. Excessive temperatures can damage cell function. We suggest using a temperature control system and recording environmental temperature changes to correct for them in subsequent analyses.
3. Mechanical stimuli (such as injections or implantation of artificial receptors) can cause local tissue damage or inflammatory responses, affecting experimental outcomes. We recommend adopting minimally invasive techniques, optimizing surgical procedures, and closely monitoring post‐operative animal behavior and physiological status.
4. Improper drug concentration and delivery methods in chemogenetic experiments can lead to non‐specific effects. We advise conducting preliminary experiments to determine optimal drug concentrations and choosing appropriate delivery methods to ensure drugs act only on target cells.
5. Changes in external magnetic fields can induce unintended neuronal responses. We recommend using shielding equipment, controlling magnetic field strength in the experimental environment, and fully considering the impact of magnetic fields during the experimental design phase.

## Conflict of Interest

The authors declare no conflict of interest.
